# Phenanthrene-like Benzodichalcogenophenes: Synthesis, Electrochemical Behavior and Applications

**DOI:** 10.3390/molecules31030425

**Published:** 2026-01-26

**Authors:** Valentina Pelliccioli, Serena Arnaboldi, Silvia Cauteruccio

**Affiliations:** Department of Chemistry, Università degli Studi di Milano, Via Golgi 19, I-20133 Milan, Italy; valentina.pelliccioli@unimi.it (V.P.); serena.arnaboldi@unimi.it (S.A.)

**Keywords:** chalcogenophene, benzodifuran, benzodithiophene, benzodiselenophene, photocyclization, Mallory reaction, Scholl reaction, organic semiconductor material, optoelectronics, shape-persistent macrocycle

## Abstract

Benzodichalcogenophenes represent a valuable class of organic π-conjugated systems that have been investigated in a plethora of cutting-edge applications in the field of materials chemistry. Isomeric benzodifuran (**BDF**), benzodithiophene (**BDT**) and benzodiselenophene (**BDS**) analogs of phenanthrene, in which the two heteroaromatic rings are *ortho*-fused onto a benzene ring, represent convenient frameworks as functional materials in organic electronics. The orientation of the two condensed heteroaromatic rings with respect to the central benzene ring provides diverse structural isomers, which significantly differ in degrees of curvature, electronic and electrochemical properties. Furthermore, tailored modification and functionalization strategies enable fine-tuning of their intrinsic properties, leading to unique systems. This review offers a comprehensive overview of synthetic methodologies for constructing isomeric **BDF**, **BDT** and **BDS** skeletons, alongside an analysis of their electrochemical properties as influenced by the nature of heteroatoms. Finally, the most relevant applications of these systems, ranging from optoelectronics, supramolecular chemistry, and emerging biological studies, are discussed, providing valuable insights for future research direction.

## 1. Introduction

Chalcogenophenes are five-membered aromatic rings incorporating a chalcogen atom (O, S and Se), known respectively as furan, thiophene and selenophene, which represent fundamental building blocks for the design of functional organic small molecules or polymer-based materials for optoelectronic applications [1,2]. Because chalcogens differ in size, polarizability, and electronegativity [3], their substitution within the chalcogenophene ring can be strategically employed to modulate photophysical properties, aromaticity, reactivity, and redox behavior of chalcogenophene-fused π-conjugated systems. The larger atomic radius and high polarizability of selenium enhance lone-pair participation in intermolecular interactions, improving charge-transport properties [4], while the smaller van der Waals radius and the higher electronegativity of oxygen promote dense packing structure and efficient carrier transport in the solid state [5]. Furthermore, furan derivatives combine strong fluorescence and high dipole moments, enabling superior solubility in polar solvents, unlike sulfur and selenium analogs whose weak fluorescence is generally attributed to the heavy-atom effect [6,7]. On the other hand, aromaticity significantly influences stability and reactivity, generally following the trend benzene > thiophene > selenophene > furan [3], although establishing a definitive ranking remains challenging [8,9]. These chalcogenophenes, being electron-rich, undergo electrophilic substitution faster than benzene, preferentially at the alpha-position (furan > selenophene > thiophene) [10,11], a trend opposite to their aromaticity due to differences in electron delocalization and orbital overlap [8,10]. Moreover, sulfur and selenium can access higher oxidation states through the involvement of d-orbitals, enabling structural modifications such as *S,S*-dioxides that profoundly alter electronic properties.

**Figure 1:**
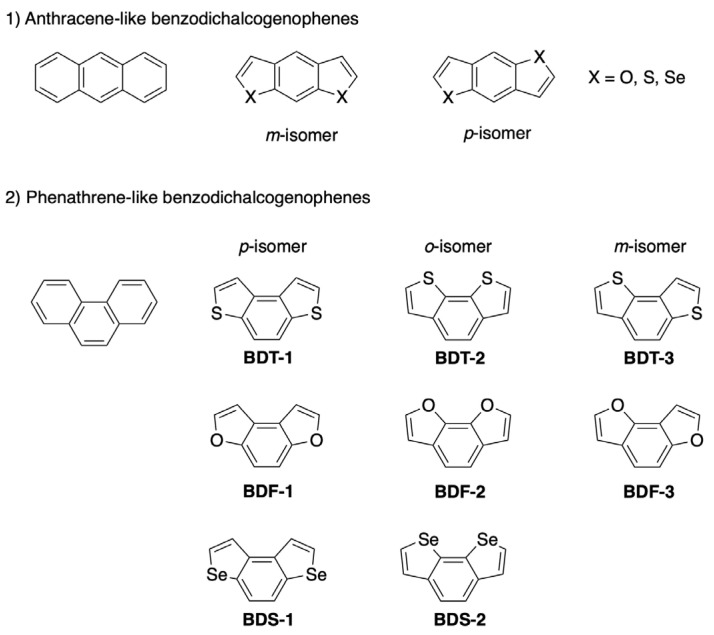
Structure of anthracene- and phenanthrene-like benzodichalcogenophenes.

The incorporation of chalcogenophenes into extended π-conjugated systems significantly affects molecular planarity, electron distribution, and intermolecular interactions. The planar structure of these compounds, which favors π–π stacking, combined with dense molecular packing and efficient charge-carrier mobility, makes them highly promising building blocks for organic electronic devices. Among these, benzodichalcogenophenes, such as benzodifurans, benzodithiophenes, and benzodiselenophenes, represent a family of tricyclic heteroaromatic compounds formed by fusing two chalcogenophene units onto a benzene ring. These systems have attracted considerable attention over the past decades for applications in organic field-effect transistors (OFETs), organic light-emitting diodes (OLEDs), and photovoltaic cells, owing to their versatile synthesis, chemical stability, and superior solubility compared to polycyclic aromatic hydrocarbon (PAH) analogs. Benzodichalcogenophenes exist as structural isomers depending on the orientation of the fused heteroaromatic rings, enabling property tuning. While structural parameters like bond length and molecular volume remain similar, their chemo-physical properties and intermolecular electronic interactions are generally different. These structural isomers are mainly divided into two linear isomers resembling the anthracene framework (Figure 1), and three angular isomers that are isoelectronic with phenanthrene and are often referred to phenanthrene-like benzodichalcogens (**BDF**, **BDT** and **BDS**, Figure 1). Despite the extensive investigation and review of linear anthracene-like isomers, especially in the context of organic electronic applications [12,13,14,15,16,17,18,19,20,21,22,23,24,25,26,27,28], to the best of our knowledge, a comprehensive discussion addressing the synthesis and major applications of phenanthrene-like counterparts **BDT**, **BDF** and **BDS** is still lacking.

This review aims to provide an overview of advances up to the end of March 2025 in the synthesis and applications of **BDT**, **BDF** and **BDS** derivatives. First, this review systematically examines synthetic strategies for constructing the tricyclic core of benzodithiophenes (i.e., benzo[1,2-*b*:4,3-*b*′]dithiophene (**BDT-1**), benzo[2,1-*b*:3,4-*b*′]dithiophene (**BDT-2**) and benzo[1,2-*b*:3,4-*b*′]dithiophene (**BDT-3**)), benzodifurans (i.e., benzo[1,2-*b*:4,3-*b*′]difuran (**BDF-1**), benzo[2,1-*b*:3,4-*b*′]dithiophene (**BDF-2**) and benzo[1,2-*b*:3,4-*b*′]dithiophene (**BDF-3**)), and benzodiselenophenes (i.e., benzo[1,2-*b*:4,3-*b*′]bis(selenophene) **BDS-1** and benzo[2,1-*b*:3,4-*b*′]bis(selenophene) **BDS-2**). Second, the electrochemical properties of **BDT**, **BDF** and **BDS** are discussed, focusing on the thermodynamic and kinetic factors that differentiate these chalcogen-fused isomers. Third, the most significant applications of these systems in optoelectronics, supramolecular chemistry, and emerging bio-related fields are reported. On the contrary, this review does not cover data reported in the patent literature or those concerning the synthesis and applications of benzotrichalcogenophenes and dione analogs (e.g., benzodifuranones and benzodithiophenone).

## 2. Synthesis of Benzodichalcogenophenes BDT, BDF and BDS

The distinct electronic properties of furan, thiophene and selenophene, together with their unique chemical behavior within the **BDF**, **BDT** and **BDS** frameworks, have driven the development of a variety of synthetic methodologies aimed at the construction of the tricyclic core. For the sake of clarity, this paragraph is organized into three parts, each addressing a specific heteroatom: beginning with **BDT** derivatives, for which the literature provides numerous examples, followed by an overview of the synthetic procedures reported for **BDF**s, and concluding with the limited procedures described to date for **BDS** systems. The discussion focuses primarily on the cyclization step leading to the formation of the tricyclic skeleton, while the preparation of the corresponding precursors is only briefly mentioned.

### 2.1. Synthesis of Benzodithiophene (**BDT**) Scaffolds

The synthesis of **BDT-1**, **BDT-2** and **BDT-3** skeleton mainly relies on the construction of the central benzene ring through three different approaches: (i) intramolecular cyclodehydrogenation of 1,2-dithienyl ethenes; (ii) intra and intermolecular annellation reactions of bithienyl derivatives; (iii) domino and/or multicomponent reactions. An alternative synthetic strategy for accessing **BDT-2** and **BDT-3** scaffolds was also proposed, based on the construction of both thiophene rings from a properly functionalized benzene core.

#### 2.1.1. Intramolecular Cyclodehydrogenation of 1,2-Dithienyl Ethenes

The intramolecular cyclodehydrogenation (ICD) of *trans* and/or *cis* isomers of 1,2-di(2-thienyl)ethenes, 1,2-di(3-thienyl)ethenes and 1,2-di(2,3′-thienyl)ethenes represented one of the first practical synthetic approaches developed for the formation of **BDT-1**, **BDT-2** and **BDT-3** skeletons, respectively. Currently, the ICD is still a very popular strategy especially to prepare **BDT-1** and **BDT-2** derivatives, due to the significant improvements achieved in the optimization of the experimental conditions along with the easy and on multigram scale syntheses of dithienyl ethenes. These latter can be generally synthesized through cheap and robust olefination procedures such as the reductive McMurry homocoupling of thienyl carboxaldehydes or ketones, especially for the preparation of symmetrical **BDT**s, and the Witting reaction and its variants to have access to symmetrical and/or unsymmetrical **BDT**s.

The ICD strategies mainly used for the synthesis of **BDT**s include the photochemically induced ICD, also known as the Mallory-type reactions, and the ICD promoted by chemical oxidants under acidic conditions through Scholl-type reactions.

##### Photochemically Induced ICD Through Mallory-Type Reaction

**Scheme 1:**
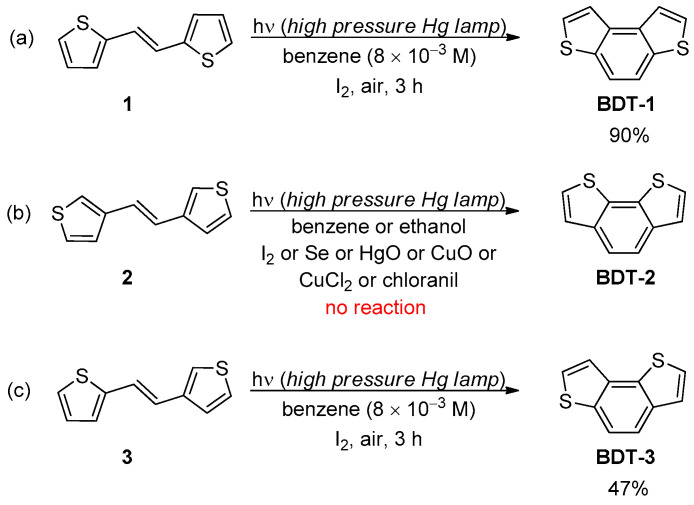
Mallory reaction for the synthesis of parent **BDT**s via photocyclization of dithienylethene **1** (**a**), **2** (**b**) and **3** (**c**).

The Mallory reaction is the photochemical cyclization of stilbene-like derivatives in the presence of catalytic amounts of iodine and is one of the most common methodologies for the synthesis of phenanthrenes, phenacenes, helicenes and other polycyclic (hetero)aromatic compounds [29,30]. This reaction proceeds through a rapid *cis*/*trans* photoisomerization of the stilbene, followed by the photo-induced cyclisation of the only *cis*-isomer and the subsequent iodine-assisted oxidation of the unstable dihydrophenanthrene intermediate in the presence of air. Thus, a *cis*/*trans* mixture of diaryl ethenes can be also used in this type of dehydrocyclization, although the yield and the reaction time are generally affected by the *cis*/*trans* ratio. The seminal work of Winberg in 1967 [31] demonstrated that the Mallory reaction could be successfully applied for the synthesis of the parent **BDT-1**, which was obtained in 90% yield by the irradiation of a solution of (*E*)-1,2-di(2-thienyl)ethene (**1**) in benzene with a high pressure mercury lamp in the presence of iodine under air for 3 h (Scheme 1a). The same reaction conditions were not as suitable for the photocyclization of ethenes **2** and **3**, indeed, while **BDT-3** was isolated in moderate yield (47%), the **BDT-2** scaffold was not obtained at all, and a complex mixture arising from polymerization processes was observed.

**Figure 2:**
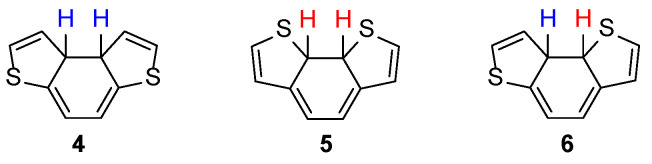
Structure of dihydro intermediates **4**–**6**.

These results were rationalized based on the different behavior of the dihydro intermediates **4**–**6** (Figure 2) in the oxidation step, in which the abstraction of one hydrogen atom is followed by a second hydrogen abstraction or β-elimination reaction.

**Scheme 2:**
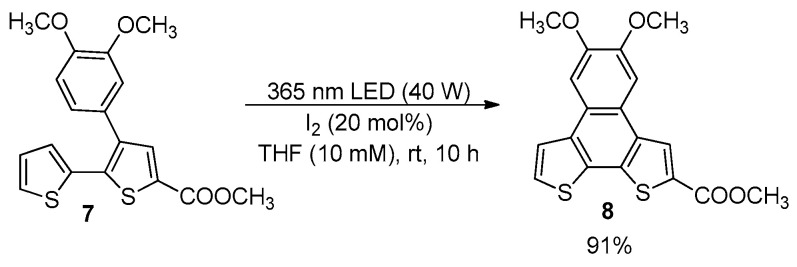
Photocyclization of dithienyl derivative **7**.

While this process was favorable for **4**, in case of **5**, the abstraction of H″ presumably resulted in the elimination of sulfur and formation of vinylthiyl radicals that in turn produced complex polymer species. For intermediate **6**, the possibility to remove H′ and H″ was the same, and then a comparable amount of **BDT-3** (47%) and polymer species were formed [32]. Recently, the photochemical cyclization towards the **BDT-2** scaffold could be efficiently accomplished starting from the 2,2′-dithienyl derivative **7**, from which the naphtho[2,1-*b*:3,4-*b*′]dithiophene **8** was isolated in 91% yield (Scheme 2) [33].

On the other hand, **BDT-1** systems functionalized with carboxylic acid [34], ester [35], phenyl [36], α-naphthyl [37] and formyl [38] groups were synthesized through oxidative photocyclizations of the corresponding dithienyl ethenes. Mallory’s conditions also afforded the **BDT-1** scaffold fused into polyaromatic hydrocarbons [39,40,41,42].

**Scheme 3:**
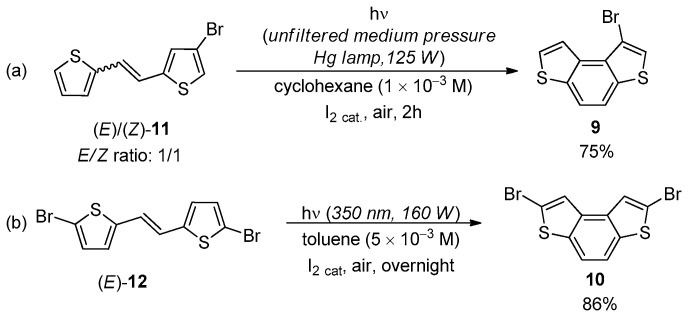
Photocyclization of bromo-substituted dithienyl ethenes **11** (**a**) and **12** (**b**).

Bromine atoms in the alpha or beta positions of the terminal thiophene rings were also well tolerated under classical Mallory conditions, and bromide **9** [43] and dibromide **10** [44] were efficiently synthesized by photocyclization of dithienyl ethenes **11** and **12** (Scheme 3).

**Scheme 4:**
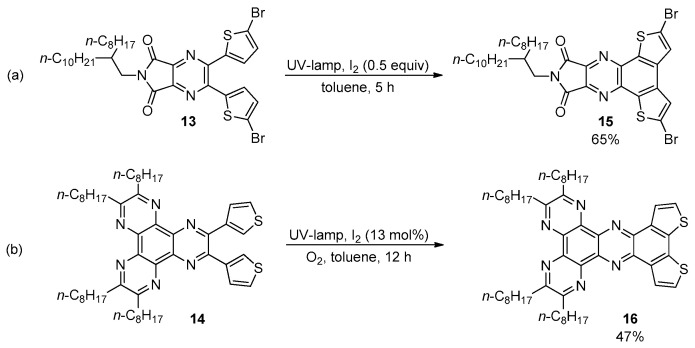
Photocyclization of pyrazine derivatives **13** (**a**) and **14** (**b**).

Mallory’s conditions were also effective in promoting the formation of **BDT-1** and **BDT-2** cores fused with nitrogen-containing heteroaromatic systems [45]. The photocyclization of pyrazines **13** [46] and **14** [47] gave the corresponding quinoxalines **15** and **16** in moderate yields (Scheme 4).

**Scheme 5:**
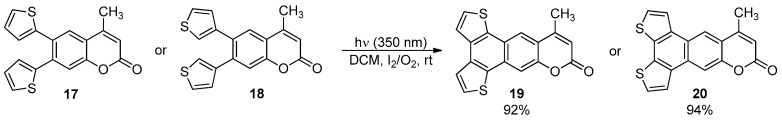
Synthesis of **BDT**-fused benzo[*g*]coumarins **19** and **20**.

More recently, the photochemical cyclization of 4-methyl-6,7-dithienylcoumarins **17** and **18** provided the corresponding **BDT-1**- and **BDT-2**-fused benzo[*g*]coumarins **19** and **20** in excellent yields (Scheme 5) [48].

**Scheme 6:**
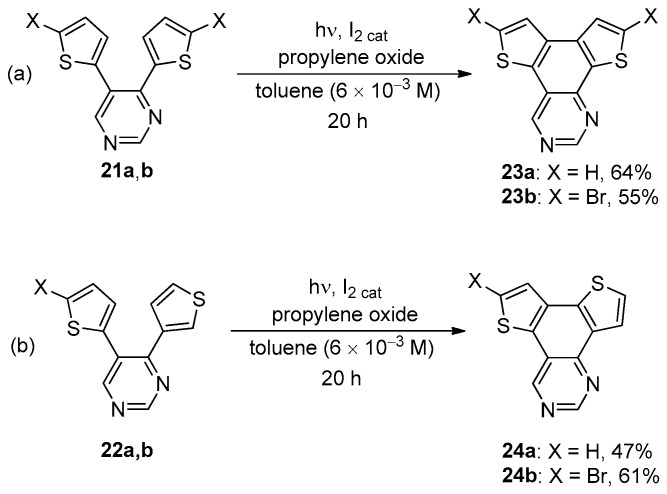
Photocyclization of 4,5-dithienyl pyrimidines **21a**,**b** (**a**) and **22a**,**b** (**b**).

The photochemical processes towards functionalized **BDT-1** and **BDT-3** were also performed in the presence of propylene oxide [49] or methyloxirane [50] as scavenger for hydrogen iodide according to Katz’s conditions [51]. These conditions also allowed photocyclizations in the presence of different heteroaromatic compounds such as 4,5-dithienyl pyrimidines **21a**,**b** and **22a**,**b**, which gave the corresponding **BDT-1** and **BDT-3**-fused systems **23a**,**b** and **24a**,**b** in moderate yields (Scheme 6) [49].

**Scheme 7:**
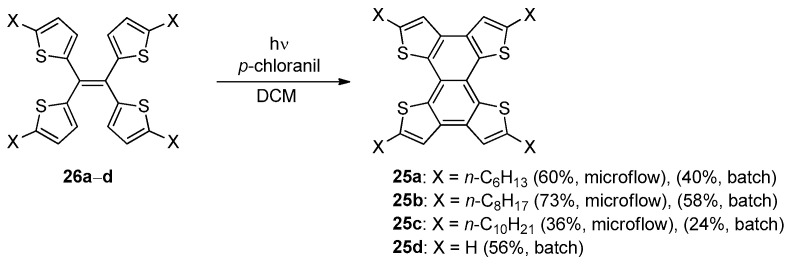
Photochemical cyclization of tetra(thien-2yl)ethenes **26a**–**d**. Batch conditions: 300 W high-pressure Hg lamp, 3–6 h of irradiation. Microflow conditions: 300 mW UV-LED lamp, 1 min of residence time, flow rate 0.4 mL/min.

The formation of a **BDT-1** core into tetrathienonaphthalenes **25** through the double photocyclization of the corresponding tetra(thien-2yl)ethenes **26** was also deeply investigated, due to their promising properties as p-type organic semiconductors [52] and for their aggregation-induced emission behavior [50]. Besides the first studies reported by Harrit in 1996 [42], alkylated tetrathienonaphthalenes **25a**–**c** were synthesized in moderate to good yields in the presence of *p*-chloranil as the electron acceptor in both batch and under continuous microflow conditions (Scheme 7) [52].

Regardless of the length of the alkyl chain, these photo-induced cyclization reactions provided products **25a**–**c** in higher yields under microflow conditions than those obtained using traditional batch reactors. The “batch” conditions also provided tetrathienonaphthalene **25d** in 56% yield, which was comparable with that obtained under the Mallory–-Katz conditions (64% [50]).

**Scheme 8:**
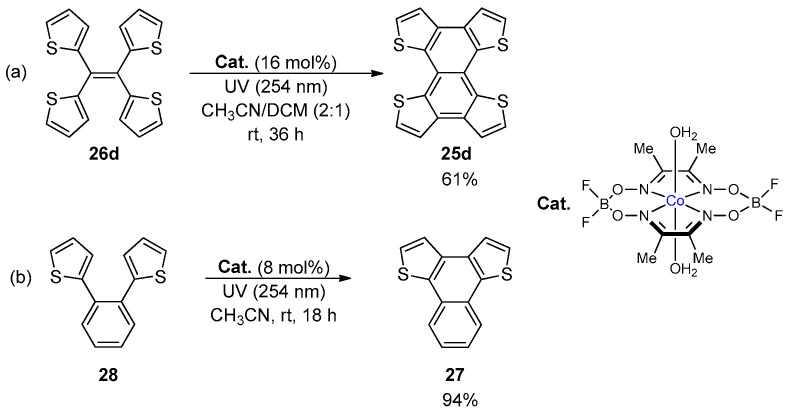
Cobaloxime-catalyzed photocyclization of **26d** (**a**) and **28** (**b**).

Alternatively, compound **25d** was also isolated in comparable yield (61%) by the cobaloxime-catalyzed dehydrogenative photocyclization of **26d** (Scheme 8a) [53]. This method represents an advantageous alternative to Mallory–Katz and Scholl reaction (*vide infra*) since it avoids the use of oxidants and strong acids. Moreover, this approach allowed the synthesis of naphtho[1,2-*b*:4,3-*b*′]dithiophene **27** from 1,2-dithienyl benzene (**28**) in excellent yield (Scheme 8b).

##### Oxidative Cyclodehydrogenation Through Scholl-Type Reaction

**Scheme 9:**
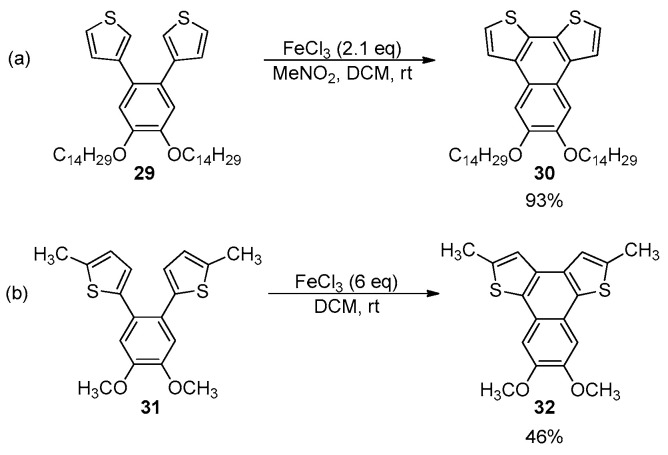
FeCl_3_-mediated cyclodehydrogenation of **29** (**a**) and **31** (**b**).

Cyclodehydrogenation promoted by organic or inorganic oxidizing agents under acidic conditions represents a complementary and effective strategy towards the formation of the **BDT-1** and **BDT-2** skeleton. At the beginning of 2000s, Tovar and Swager [54,55] reported an in-depth study on the chemical and electrochemical oxidation of 1,2-bis-(2-thienyl) and 1,2-bis-(3-thienyl)benzene derivatives, and in particular the FeCl_3_-mediated oxidative cyclization of 1,2-bis-(3-thienyl)benzene derivative **29** provided the corresponding naphtho[2,1-*b*:3,4-*b*′]dithiophene **30** in high yield and selectivity (Scheme 9a) [54].

A large molar excess of the oxidant allowed the complete conversion of the starting materials, and any significant polymerization by-products were observed. Otherwise, the formation of the carbon–carbon bond at the β-positions of 1,2-bis-(2-thienyl)benzene **31** to obtain the naphtho[1,2-*b*:4,3-*b*′]dithiophene **32** required the protection of the α-positions of thiophene rings to form the desired **BDT-1** framework and prevented polymerization processes due to the high spin density of the thiophene radical cation at the 2-position (Scheme 9b).

After this study, the Scholl-type reaction [56] was extensively investigated for intramolecular oxidative C–C bond formation between two thienyl rings to yield **BDT**-based π-conjugated materials. The latter make use of FeCl_3_ or MoCl_5_ in dichloromethane or organic oxidants, such as dichlorodicyano-*p*-benzoquinone (DDQ), chloranil or bis(trifluoroacetoxy)iodobenzene (PIFA), in combination with strong Lewis or Brønsted acids. Although the mechanism of Scholl’s reaction is still controversial, the arenium cation and the radical cation mechanism are the most accepted, and both require that the two thienyl rings rely on the same side to form the biaryl linkage. Thus, only *cis*-1,2-dithienyl ethenes or 1,2-dithienyl benzene derivatives are suitable substrates for this reaction, and they can be prepared through stereoselective olefination reactions (i.e., McMurry coupling of ketones) or palladium-catalyzed cross coupling reactions (i.e., Stille and Suzuki coupling) between aryl halides and thiophene-based organostannane or organoboron compounds. On the other hand, the FeCl_3_-mediated cyclodehydrogenation was found to be a more effective strategy for the construction of the **BDT-2** skeleton in comparison with the photochemical approach, and allowed for obtaining the **BDT-2** core incorporated into naphthodithiophenes [57], tetrathienoanthracenes [58], dithieneonaphthothiadiazoles [59,60], porphyrins [61], and anthracene-, pyrene- and perylene-base systems [62].

**Scheme 10:**
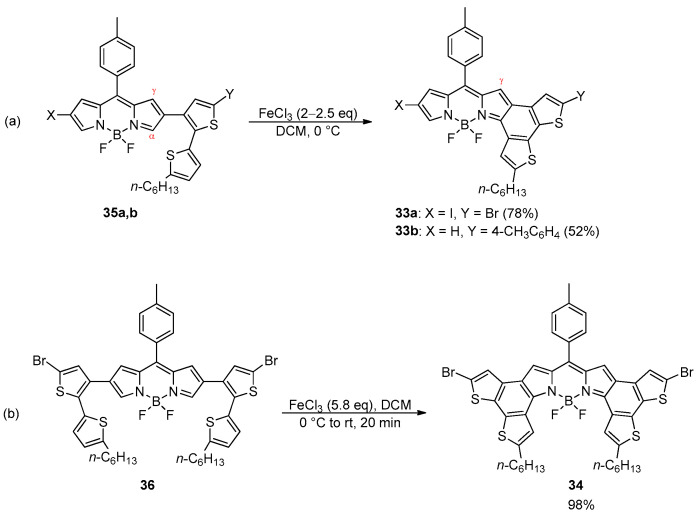
Synthesis of **BDT-2**- fused BODIPY dyes **33a**,**b** (**a**) and **34** (**b**).

Notably, the formation of the **BDT-2** core to yield the corresponding α-fused BODIPY dyes **33a**,**b** and **34** was achieved through the selective oxidative coupling of dithienyl derivatives **35a**,**b** and **36** in the presence of anhydrous FeCl_3_ (Scheme 10) [63].

The α-position of the BODIPY dyes was found to be the most reactive site toward the intramolecular oxidative reaction, providing the γ-fused forms as the main products.

The formation of the **BDT-1** scaffold could also be easily accomplished by the oxidative FeCl_3_-, MoCl_5_- or DDQ-mediated cyclodehydrogenation of 1,2-dithienyl benzenes or more complex systems to synthesize naphthodithiophenes [64,65,66], dithieneonaphthothiadiazole [60], tetrathienoanthracenes [67,68,69], pyrene- [40,70] chrysene- [71] and triphenylene-based systems [72], benzo[*k*]fluoranthene derivatives [73], tetracene diimide [74], functionalized imides [75,76], benzoquinones [77], extended tetracyano-*p*-quinodimethane [78], highly π-conjugated quinacridones [79], and tetrathienodiborapentacenes [80].

**Scheme 11:**
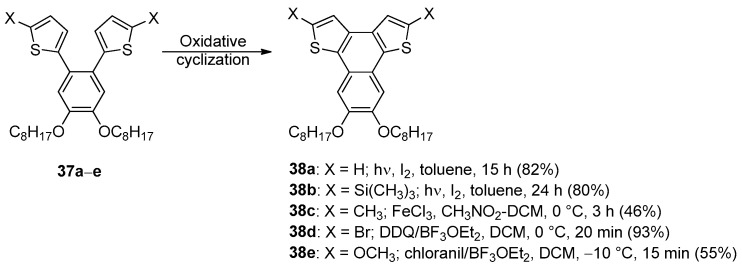
Oxidative cyclization of bis(2-thienyl) benzenes **37**.

In 2013, a comprehensive study on the intramolecular cyclodehydrogenation of functionalized 1,2-bis(octyloxy)-4,5-bis(2-thienyl)benzenes **37** towards the synthesis of the corresponding naphthothiophenes **38** showed how the nature of the substituents in the alfa positions of the thiophene rings in combination with the nature of the chemical oxidant affected the outcome of the oxidative cyclization (Scheme 11) [66].

Unsurprisingly, the selective intramolecular cyclization of substrates **37a**,**b** could be achieved only under oxidative photochemical conditions, since the use of FeCl_3_ in nitromethane or DDQ-BF_3_•OEt_2_ at 0 °C gave polymerization and deprotection followed by polymerization, respectively, after 20 min.

**Scheme 12:**
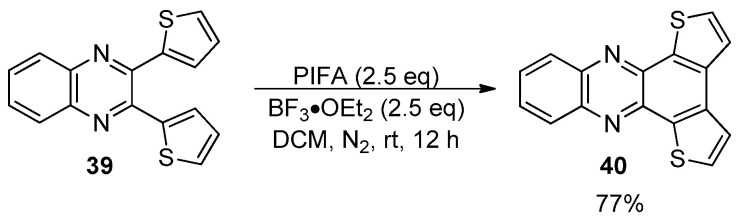
PIFA-mediated cyclodehydrogenation of **39**.

Although the Scholl reaction has been predominantly applied to electron-donating systems, examples involving electron-withdrawing heterocycles, while less common, are also reported [81,82,83]. The PIFA-BF_3_•OEt_2_-mediated oxidative cyclodehydrogenation of dithienyl quinoxaline **39** provided the corresponding phenazine **40** in good yield in the presence of the free alfa positions of the thiophene rings (Scheme 12) [82].

**Scheme 13:**
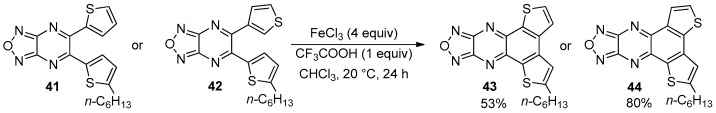
FeCl_3_-mediated cyclodehydrogenation of **41** and **42**.

More recently, the formation of **BDT-1** and **BDT-3** skeletons was promoted by the FeCl_3_-mediated oxidative cyclodehydrogenation of *ortho*-dithienyl substituted furazanopyrazines **41** and **42**, from which the corresponding polycyclic systems **43** and **44** were isolated in moderate yields (Scheme 13) [83].

**Scheme 14:**
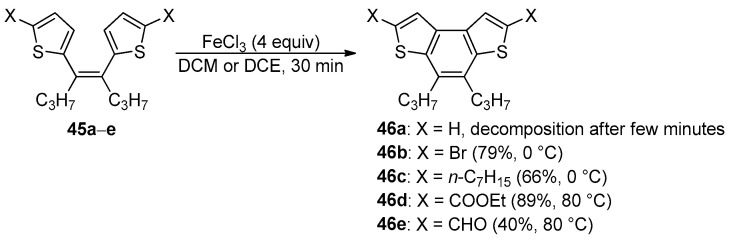
FeCl_3_-mediated cyclization of (*Z*)-dithienyl ethenes **45**.

While the oxidative cyclodehydrogenation was mainly applied on 1,2-dithienyl benzene derivatives, only one example of cyclization of *cis*-1,2-dithienyl ethenes was described [84]. The FeCl_3_-mediated cyclization of (*Z*)-dithienyl ethenes **45** was reported for the synthesis of functionalized 4,5-dipropylbenzodithiophenes **46** (Scheme 14) [84].

The nature of the substituents in the alfa positions and the reaction temperature significantly affected the efficacy and selectivity of this reaction: (i) the complete decomposition of the alkene without α-substituents **45a** was observed at room temperature or 0 °C; (ii) the cyclization of dibromo and dialkyl alkenes **45b**,**c** was favored by low temperatures; (iii) the alkenes bearing electron-withdrawing groups **45d**,**e** required higher temperatures (up to 80 °C) for the formation of the corresponding **BDT-1** derivatives [84].

#### 2.1.2. Intramolecular and Intermolecular Annellation Reactions of Bithienyl Derivatives

Since the first decade of the 2000s, an alternative approach to the cyclodehydrogenation reactions of dithienyl alkenes or benzene derivatives was explored and applied for the synthesis of **BDT-1**, **BDT-2** and **BDT-3**, and it involves the construction of their central benzene ring via intra- and intermolecular annellation reactions of the corresponding functionalized 3,3′- and 2,2′-bithienyl compounds.

##### Intramolecular Annellations

The 3,3′-bithienyl derivatives, properly modified in the alpha positions with carbonyl and/or alkenyl pendants, are readily available by common homo-coupling reactions, and they represent key intermediates to forge the benzene ring of the **BDT-1** derivatives through intramolecular McMurry coupling [85,86], the ring-closing metathesis (RCM) [87], and the ring-closing carbonyl–olefin metathesis (RCCOM) [88].

**Scheme 15:**
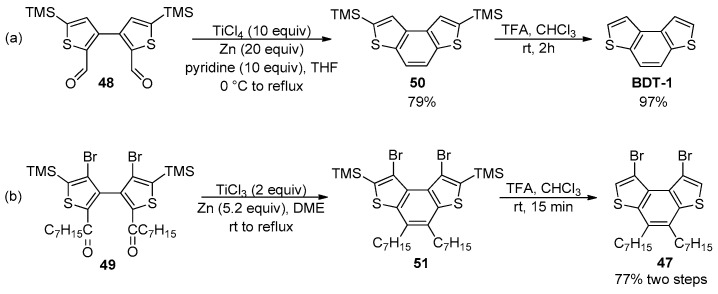
Intramolecular McMurry coupling for the synthesis of **BDT-1** (**a**) and dibromide **47** (**b**).

The synthesis of the parent **BDT-1** and the dibromide **47** could be achieved by a two-step procedure involving the intramolecular McMurry coupling of dialdehyde **48** [85] and diketone **49** [86], respectively, followed by the smooth and efficient removal of both trimethylsilyl (TMS) groups from intermediates **50** and **51** under acidic conditions (Scheme 15).

**Scheme 16:**
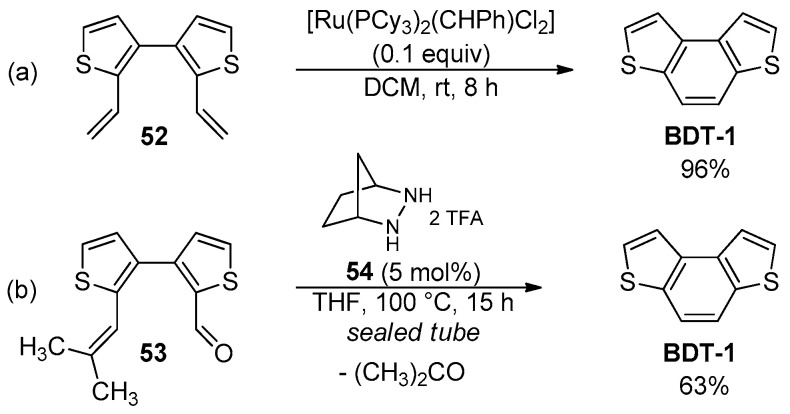
Synthesis of the parent **BDT-1** via: (**a**) RCM reaction; (**b**) metal-free RCCOM.

On the other hand, the RCM reactions of 2,2′-divinylbiphenyl derivatives have been extensively used to prepare phenanthrene-like compounds, including the parent **BDT-1**, which was obtained in high yield by the Ru-catalyzed RCM of 2,2′-divinyl-3,3′-bithiophene **52** using the 1st generation Grubbs catalyst (Scheme 16a) [87].

The RCCOM reaction has also proven suitable for the synthesis of a variety of polycyclic heteroaromatic molecules [89], and a metal-free RCCOM protocol [88] promoted by strained hydrazines as organocatalysts was recently developed to prepare functionalized benzo[*h*]isoquinolines, naphthofurans and thiophene-containing systems such as the parent **BDT-1**. This latter was isolated in 63% yield by the condensation of the aldehyde **53** in the presence of dialkylhydrazine salt **54** in THF at 100 °C, obtaining acetone as the easily removable waste product (Scheme 16b).

**Scheme 17:**
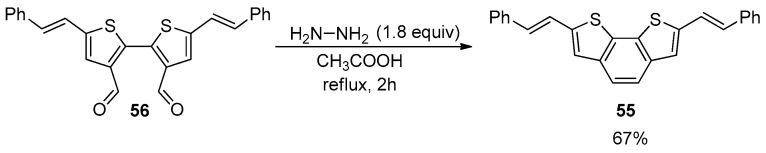
Reductive coupling of dialdehyde **56** towards **BDT-2** system **55**.

The intramolecular reductive coupling of a 2,2′-bithienyl derivative bearing two formyl groups in the beta positions for the formation of the **BDT-2** skeleton was also reported (Scheme 17) [90].

Product **55** was isolated in 67% yield by the hydrazine-mediated cyclization of dialdehyde **56** in acetic acid at reflux, according to the standard Bacon’s conditions [91]. It should be noted that the reductive coupling from bis-tosylhydrazones of 3,3′-bithienyls towards the formation of **BDT-1** derivatives was also explored according to Jung’s conditions [92], though low yields were achieved (32–37%) [87].

**Scheme 18:**
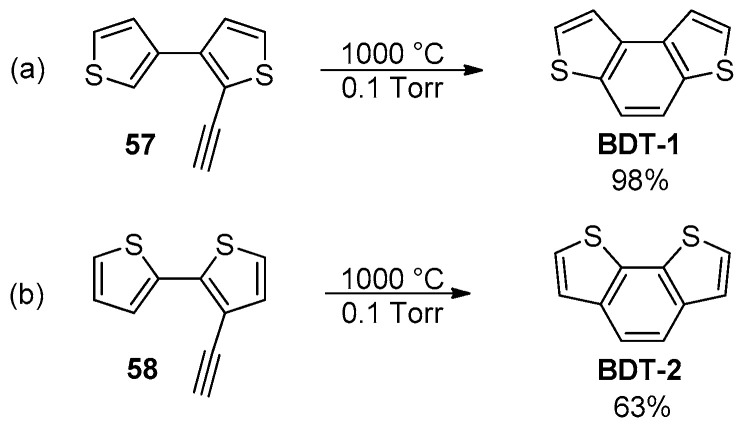
FVP for the synthesis of parent **BDT-1** (**a**) and **BDT-2** (**b**).

2-Ethynylbithienyl systems are equally useful and easily accessible intermediates, from which the **BDT** skeleton can be formed through thermal-induced cyclizations as well as cycloisomerization reactions triggered by electrophilic reagents such as Lewis acids or metal complexes. First studies on the conversion of 2-ethynyl-3,3′-bithiophene (**57**) and 3-ethynyl-2,2′-bithiophene (**58**) into the parent **BDT-1** and **BDT-2**, respectively, were reported using the flash vacuum pyrolysis (FVP) (Scheme 18) [93].

**Scheme 19:**
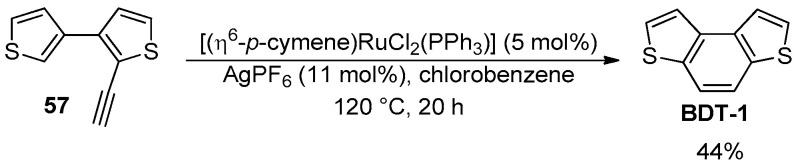
Ru-catalyzed cycloisomerization of **57**.

Benzodithiophenes were isolated in good to excellent yields (63–98%) and with complete regioselectivity for **BDT-1**, although this approach has not had further synthetic applications in this context. Otherwise, since the seminal work of Fürstner and co-workers [94], the intramolecular hydroarylation of 2-alkynylbiaryls mediated by metal catalysts or Lewis acid organocatalysts under much milder conditions has been applied for the synthesis of a great variety of phenanthrene-like derivatives and larger poly(hetero)aromatic molecules [95]. This approach was also applied for the synthesis of the **BDT-1**, which was isolated in moderate yield by the Ru-catalyzed cycloisomerization of 2-ethynyl-3,3′-bithiophene (**57**) (Scheme 19) [96].

**Scheme 20:**
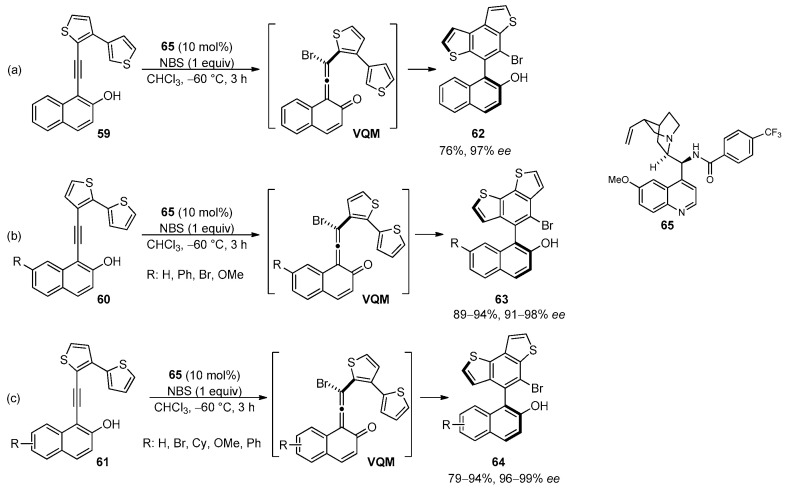
Atroposelective synthesis of naphthyl-based **BDT** derivatives **62** (**a**), **63** (**b**) and **64** (**c**).

More recently, properly designed 3,3′-, 2,2′- and 3,2′-bithienyls **59**, **60** and **61** were employed for the efficient and atroposelective synthesis of axially chiral naphthyl-based **BDT** derivatives **62**, **63** and **64** (Scheme 20) [97].

The reaction proceeded through an intramolecular 6π-electrocyclization of the vinylidene *ortho*-quinone methide (**VQM**) intermediate with the thiophene ring, in the presence of *N*-bromosuccinimide (NBS) as brominating electrophile and the quinine-derived amide **65** as chiral Brønsted base organocatalyst. This procedure was also successfully applied for the synthesis of axially chiral naphthyl-based **BDF-1** derivative (90% yield, 93% *ee*) and thieno[3,2-*e*]benzofurans (up to 97% yield, 96% *ee*) [97].

**Scheme 21:**
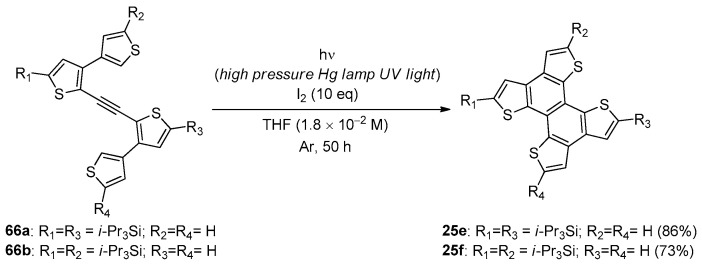
Photocyclization of bis(bithienyl)acetylenes **66** towards tetrathienonaphthalenes **25e**,**f**.

The chemistry of the intramolecular annellation of alkynylated biaryls was also applied by Yamaguchi et al. [98] for the effective one-pot synthesis of tetrathienonaphthalenes **25e**,**f**, which were obtained by irradiation of a THF solution of bis(bithienyl)acetylenes **66a**,**b** with a high-pressure mercury lamp in the presence of a large molar excess of I_2_ (Scheme 21).

**Scheme 22:**
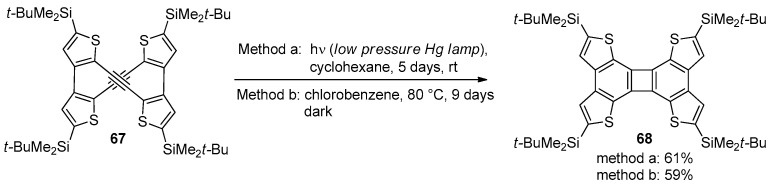
[2+2]-Cycloaddition of alkyne **67** for the synthesis of cycloadduct **68**.

The formation of the corresponding 6-*endo* cyclized products, **25e**,**f**, presumably occurred through a one-pot process that involved an iodine-promoted electrophilic monocyclization, followed by a second UV-induced electrocyclization and aromatization. The same authors described the synthesis and the intriguing reactivity of the thiophene-containing bisdehydro[12]annulene **67**, which was able to undergo a metal-free [2+2]-type alkyne cycloaddition under either photoirradiation or mild thermal conditions (Scheme 22) [99].

The possibility of obtaining the **BDT**-based cycloadduct **68** with mild heating is likely ascribed to the less aromatic character of thiophene, which was more prone to losing aromaticity during the thermal process.

##### Intermolecular Annellations

**Scheme 23:**
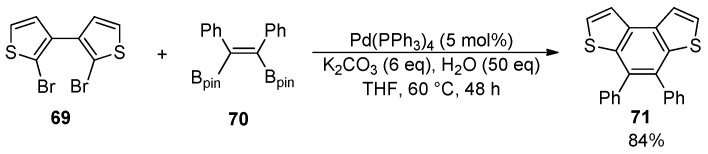
Double Suzuki coupling for the synthesis of 4,5-diphenyl substituted **BDT-1 71**.

A typical example of intermolecular annellation for building polycyclic (hetero)aromatic compounds makes use of palladium-catalyzed cross-coupling reactions, including the Suzuki and the Stille coupling, which were also employed for the formation of the central benzene ring of the **BDT-1** and **BDT-2** skeleton. The double Pd(0)-catalyzed Suzuki coupling between dibromide **69** and the (*Z*)-1,2-bis(pinacolatoboryl)stilbene **70** provided the disubstituted **BDT-1** derivative **71** in 84% yield (Scheme 23) [100].

**Scheme 24:**
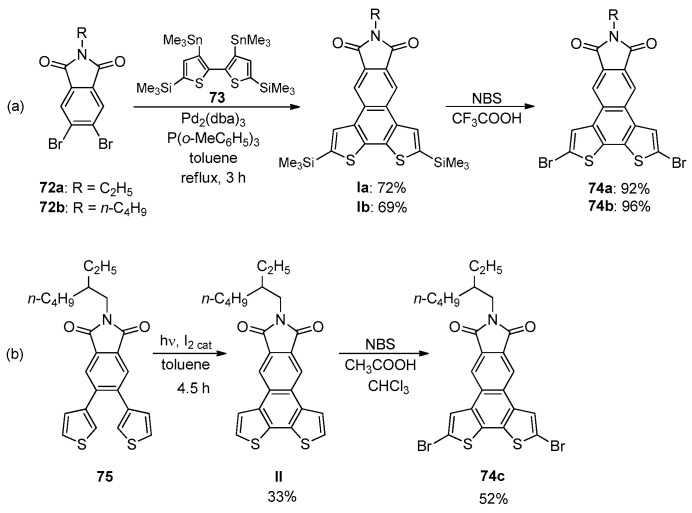
Formation of **BDT-2** skeleton in bromides **74** via: (**a**) double Stille coupling reaction between **72a**,**b** and **73**; (**b**) Mallory-type photocyclization of **75**.

Compound **71** was also obtained in 70% yield through the photocyclization of 1,2-diphenyl-1,2-di(thiophene-2-yl)ethene under classical Mallory conditions [36]. The double Pd(0)-catalyzed Stille coupling between the dibromobenzene imides **72a**,**b** and 2,2-bithienyl distannane **73**, followed by the one-pot process involving the removal of the TMS groups and bromination, gave the **BDT-2** bromides **74a**,**b** in good yields (Scheme 24a) [101].

This two-step procedure for the synthesis of bromides **74** was more efficient than that of the construction of the **BDT-2** accomplished by Mallory-type photocyclization of **75** (Scheme 24b) [102].

**Scheme 25:**
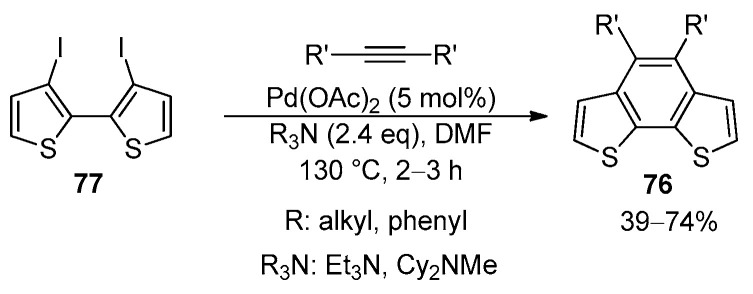
Intermolecular Pd-catalyzed annulation towards disubstituted **BDT-2** derivatives **76**.

The synthesis of 4,5-disubstituted **BDT-2** derivatives **76** was also reported via the intermolecular Pd(OAc)_2_-catalyzed annulation of 3,3′-diiodo-2,2′-bithiophene (**77**) with internal alkynes in the presence of a trialkyl amine in DMF at 100–130 °C under ligandless conditions (Scheme 25) [103,104].

**Scheme 26:**
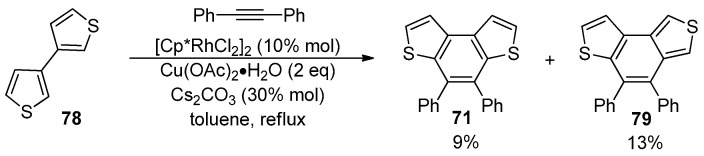
Rh-catalyzed dehydrogenative coupling of **78** with diphenylacetylene.

Besides the palladium chemistry, the Rh(III)-catalyzed dehydrogenative coupling of heterobiaryls with alkynes represents a convenient alternative towards the synthesis of fused polycyclic heteroarenes [105]. This approach does not require a pre-functionalization of the biaryl systems since it generally proceeds via a direct C–H bond cleavage followed by the alkyne insertion and reductive elimination. Until now, attempts to obtain the **BDT** skeleton through this approach have not provided satisfactory results, such as the rhodium-catalyzed annulation of 2,2′-bithiophene (**78**) with diphenylacetylene, from which a mixture of 4,5-diphenyl substituted **BDT** systems **71** and **79** was obtained in very low yield (Scheme 26) [106].

**Scheme 27:**
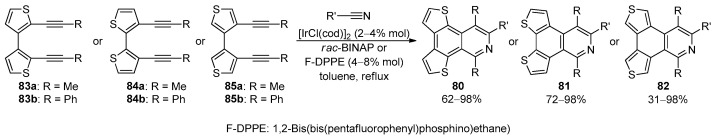
Synthesis of **BDT**-fused isoquinolines **80**–**82** through Ir-catalyzed [2+2+2]-cycloaddition of nitriles.

More recently, different **BDT**-fused isoquinolines **80**–**82** were obtained in good to excellent yield by the iridium-catalyzed [2+2+2] cycloaddition of nitriles with bithiophenes **83**–**85** bearing two alkynyl pendants (Scheme 27) [107].

The metal-catalyzed [2+2+2] cycloaddition reaction is indeed an atom-economical approach for the formation of several carbon–carbon and carbon–heteroatom bonds in one step and allows for straightforward access to complex polycyclic (hetero)aromatic compounds [108]. The scope of this reaction was deeply investigated, demonstrating the high versatility and efficacy of this approach, especially towards isoquinolines **80** and **81**.

#### 2.1.3. Domino and Multicomponent Reactions

**Scheme 28:**
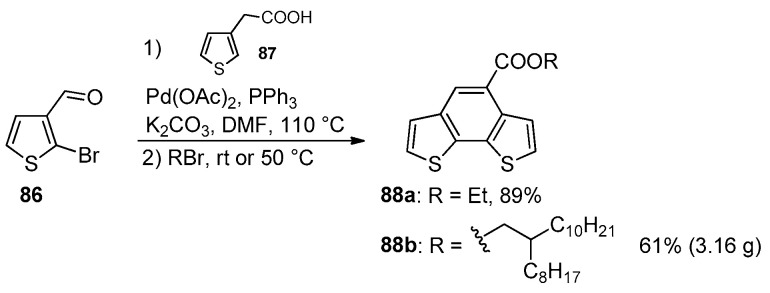
Domino one-pot synthesis of **BDT-2** derivatives **88a**,**b**.

The atom-economical domino and multicomponent reactions have proven to be a convenient and alternative route to access **BDT** derivatives. In this context, the regioselective construction of the **BDT-2** scaffold could be achieved by a domino one-pot protocol involving the direct arylation reaction and a cross-aldol intramolecular condensation starting from readily available reagents **86** and **87** (Scheme 28) [109,110].

The **BDT-2** derivative **88a** was isolated in high yield, and the presence of the electron-withdrawing carboxylate ester was found to be essential in favoring the intramolecular cross-aldol condensation [109]. This protocol was also applied for the synthesis on a gram-scale of a **BDT-2** derivative **88b** bearing a long alkyl chain to improve its solubility in common organic solvents and to then facilitate its usage in organic devices [110].

**Scheme 29:**
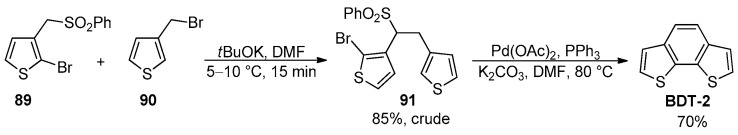
Domino reaction towards the parent **BDT-2**.

A complementary strategy to build the parent **BDT-2** made use of a two-step reaction involving the alkylation at low temperature of 2-bromo-3-(phenylsulfonylmethyl)thiophene (**89**) with 3-bromomethylthiophene (**90**), followed by the intramolecular direct arylation of sulfone **91** and the concomitant elimination of phenylsulfinic acid to yield the aromatic structure (Scheme 29) [111].

**Scheme 30:**
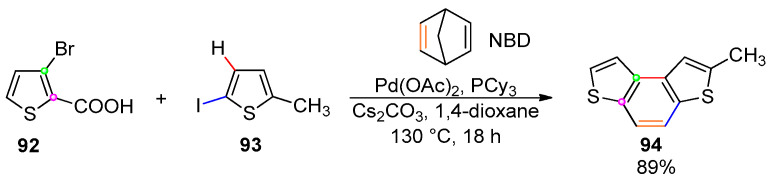
NBD-mediated palladium cascade reactions towards **94**.

An efficient and highly regioselective multicomponent approach towards π-extended (hetero)aromatic compounds involves the use of norbornadiene (NBD) as a key building block in the palladium-catalyzed C–H functionalization of (hetero)aryl halides, where the NBD derivatives act as an *ortho*-C–H activator and ethylene synthon [112]. As far as the **BDT** skeleton, the palladium-catalyzed three-component reaction between the easily available thienyl derivatives **92** and **93** and NBD provided the substituted **BDT-1 94** in good yield (Scheme 30).

DFT calculations suggested that the reaction occurred via the decarboxylative NBD-mediated palladium cascade, in which the Pd(II)-Pd(IV) oxidative addition seemed to be the rate-determining step, followed by the retro-Diels-Alder (rDA) reaction of the NBD-fused intermediate to yield the phenanthrene-like **BDT-1** scaffold.

**Scheme 31:**
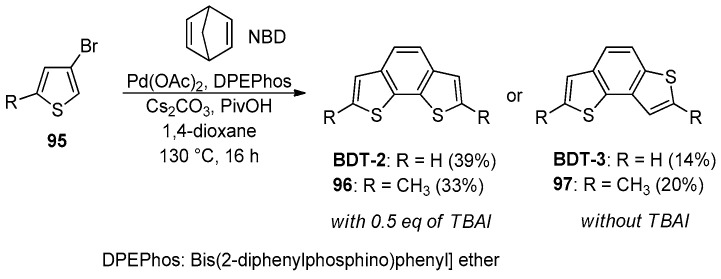
NBD-mediated palladium cascade reactions towards **BDT-2** and **BDT-3** cores.

The palladium-catalyzed multicomponent coupling reaction of bromothiophenes **95** and NBD, followed by rDA via a 2:1 annulation, was also employed to build the **BDT-2** and **BDT-3** frameworks (Scheme 31) [113].

Tetrabutylammonium iodide (TBAI) significantly affected the selectivity of this reaction towards the *cis*- and *trans*-annulation, though for both **BDT** derivatives, low yields were achieved.

**Scheme 32:**
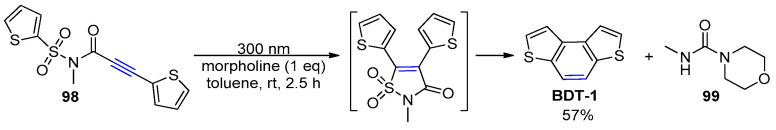
Synthesis of **BDT-1** through one-pot Smiles rearrangement-Mallory photocyclization.

Finally, a one-pot process involving the *N*-methyl-3-(2-thienyl)-*N*-(2-thienylsulfonyl)propiolamide **98** was reported for the synthesis of the parent **BDT-1** (Scheme 32) [114].

The base-assisted photoinduced reaction of a toluene solution of **98** afforded **BDT-1** in 57% yield via the one-pot Smiles rearrangement and Mallory-type oxidative cyclization, followed by the removal of the sulfonylamide moiety. This latter step was proven to occur through a ring-opening reaction followed by elimination of an isocyanic intermediate and its trapping by morpholine to yield morpholine-4-carboxamide **99**.

#### 2.1.4. Construction of Both Thiophene Rings from Arylethynylated Naphthalenes

**Scheme 33:**
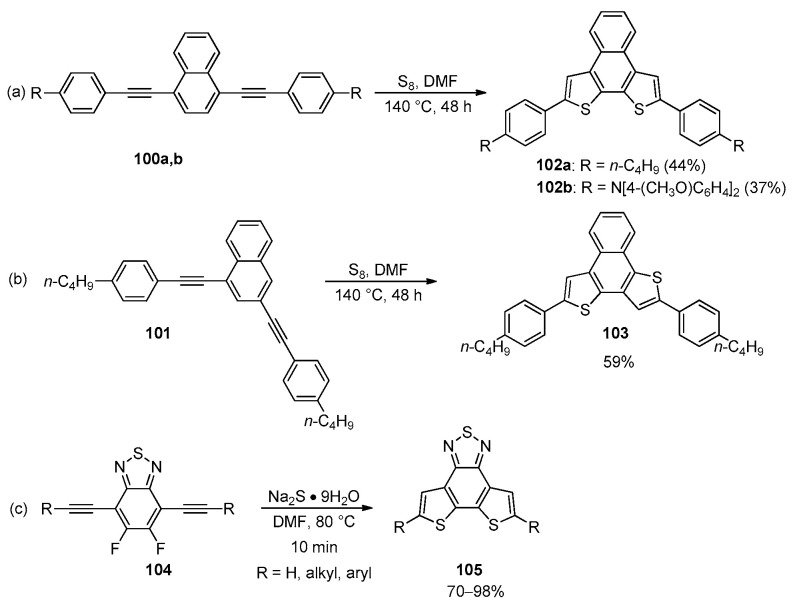
Thienannulation of arylethynylated naphthalenes **100** (**a**), **101** (**b**) and ethynylbenzenes **104** (**c**).

The metal-free thienannulation of arylethynylated naphthalenes **100a**,**b** and **101**, via *ortho*-C-H bond cleavage promoted by elemental sulfur in DMF at 140 °C, provided the corresponding naphtho[2,1-*b*:3,4-*b*′]dithiophene **102a**,**b** and naphtho[1,2-*b*:3,4-*b*′]dithiophene **103** in moderate yields (Scheme 33a,b) [115,116].

In addition, the reaction of 2-fluoroethynylbenzenes **104** with Na_2_S as nucleophile efficiently led to the formation of the **BDT-2** skeleton in dithienobenzothiadiazoles **105** via a cascade sequence involving nucleophilic aromatic substitution followed by anionic cyclization onto the pendant alkyne (Scheme 33c) [117]. Remarkably, this thiolation annulation proceeds without any transition-metal catalyst and in excellent yields within 10 min.

### 2.2. Synthesis of Benzodifuran (**BDF**) Scaffolds

**Scheme 34:**
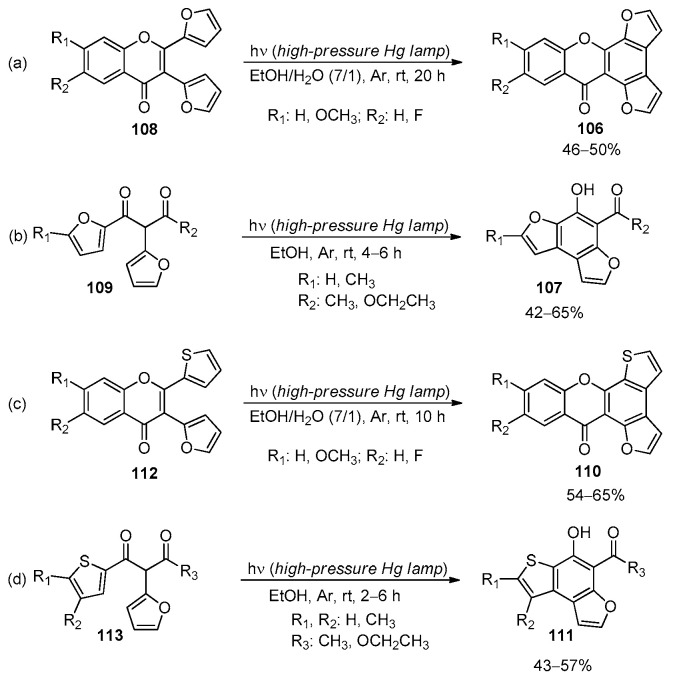
Oxidative photocyclization towards the synthesis of derivatives **106** (**a**), **107** (**b**), **110** (**c**) and **111** (**d**).

The earliest approach to the preparation of parent **BDF-1** involved constructing the central benzene ring via the oxidative photochemical cyclization of difurylethene [31]. Although this method is widely used for the synthesis of **BDT** cores, its use for **BDF** has remained limited. Notably, in 2017, the formation of the central benzene ring of **BDF-1** skeleton in derivatives **106** [118] and **107** [119] was accomplished by photoinduced oxidative cyclization in a polar protic solvent (EtOH), without the need for any oxidant and catalyst (Scheme 34a,b).

**BDF-1** derivatives **106** were obtained in moderate yields by the direct intramolecular annulation of 2,3-di(fur-2-yl)arylchromen-4-ones **108** in a mixture of water and EtOH at room temperature (Scheme 34a), while the photocyclization of **109** under similar reaction conditions provided furan[3,2-*e*]benzofurans **107** in 42–65% yields by a photoinduced process involving keto-enol tautomerization steps (Scheme 34b). These synthetic methodologies also enabled the preparation of related tricyclic analogs, such as thieno[3,2-*e*]benzofuran **110** and **111** (Scheme 34c,d), which were obtained through the photocyclization of **112** and **113**, respectively, in yields comparable to those of the corresponding **BDF-1** derivatives **106** and **107**.

Aside from these examples, the formation of **BDF-1**, **BDF-2** and **BDF-3** cores mainly relies on three strategies: (i) construction of both furan rings starting from functionalized benzenes; (ii) formation of a furan ring from suitably modified benzofurans; (iii) formation of a benzofuran core from furan-bridged enynes.

#### 2.2.1. Construction of Both Furan Rings from Functionalized Benzenes

**Scheme 35:**
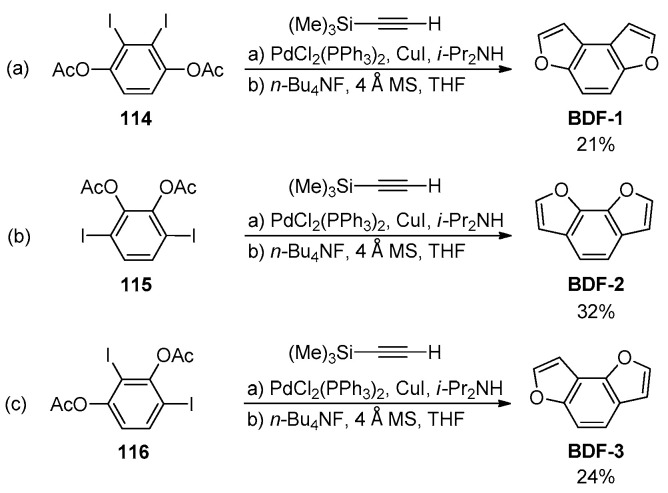
Synthesis of parent **BDFs** through cyclization of modified hydroquinones **114** (**a**), **115** (**b**) and **116** (**c**).

Properly designed hydroquinones can serve as versatile starting materials for the synthesis of various isomeric benzodifurans. Parents **BDF-1**, **BDF-2** and **BDF-3** could be obtained through a Sonogashira coupling between trimethylsilylacetylene and diester of diiodohydroquinone **114**, diiodocatechol **115** and diiodoresorcinol **116**, respectively, followed by a sequential cyclization and desilylation reactions promoted by tetrabutylammonium fluoride (Scheme 35) [120].

**Scheme 36:**
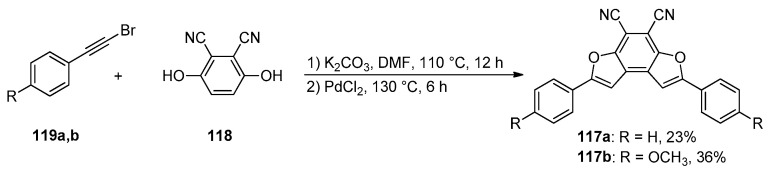
Synthesis of **BDF-1** derivatives **117a**,**b**.

The same approach was also employed for the synthesis of functionalized **BDF-3** scaffolds [121]. Alternatively, **BDF-1** systems **117a**,**b** could be accessed through one-pot reactions involving the addition of hydroquinone **118** to bromoalkynes **119a**,**b**, followed by the intramolecular cyclization via palladium-catalyzed C–H bond functionalization (Scheme 36) [122].

This methodology [123], which does not require *ortho*-halogenated phenols, represents a valid alternative to the Sonogashira coupling/cyclization strategy discussed above and provides the final **BDF-1** systems in similar yields to those reported in Scheme 35.

**Scheme 37:**
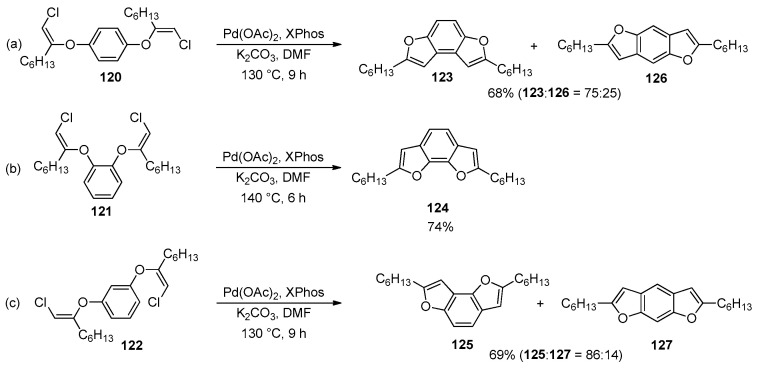
Double C−H cyclization of chloroolefins **120** (**a**), **121** (**b**) and **122** (**c**) for the synthesis of alkylated **BDF** derivatives **123**–**127**.

More recently, chloroolefins **120**, **121** and **122**, obtained via selective nucleophilic addition of hydroquinone, resorcinol, and catechol, respectively, to 1-chloro-1-octyne, have proven to be suitable intermediates for the synthesis of the corresponding alkylated **BDFs** systems **123**–**125** (Scheme 37) [124].

This transformation proceeds via an intramolecular palladium-catalyzed C–H cyclization carried out using a catalytic system made of Pd(OAc)_2_ and 2-dicyclohexylphosphino-2′,4′,6′-triisopropylbiphenyl (XPhos). The cyclization of **120** and **122** proceeded with no complete selectivity due to concurrent dual C–H cyclization within the same molecules. Thus, regioisomers **123** and **126** were isolated as a mixture (75/25 ratio) in 68% yield from chloroolefin **120**, while **125** and **127** were obtained as a mixture in a similar ratio and yield from chloroolefin **122** (Scheme 37a,c). The **BDF-2** derivative **124** was isolated as a single isomer in 74% yield, presumably because of the limited number of activatable C−H bonds (Scheme 37b).

**Scheme 38:**
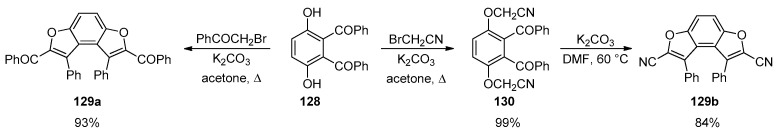
Synthesis of **BDF-1** derivatives **129a**,**b** via alkylation/cyclization.

Substituted hydroquinone **128** was used to prepare **BDF-1** derivatives **129a**,**b** in high yields via alkylation and dehydrative cyclization reactions (Scheme 38) [125].

**Scheme 39:**
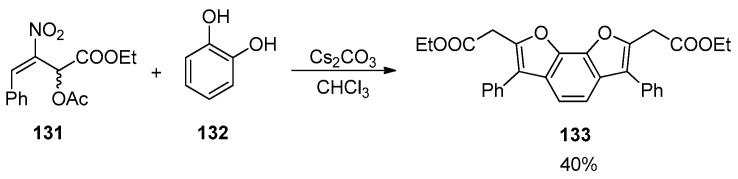
Synthesis of **BDF-2** derivative **133**.

Notably, for the compound **129a**, the alkylation and cyclization/dehydration were accomplished in a one-pot process under refluxing acetone. A similar strategy was also employed for the preparation of difuro[2,3-*e*:2, 3-G]indoles [126]. Conversely, a Cs_2_CO_3_-promoted substitution-elimination process between nitroallylic acetate **131** with catechol (**132**) gave the corresponding **BDF-2** derivative **133** in moderate yield (Scheme 39) [127].

A reasonable mechanism for this transformation involves a Friedel–Crafts-type reaction through an S_N_2′ process, followed by an intramolecular oxa-Michael cyclization and the subsequent energetically favored aromatization of the furan cores.

**Scheme 40:**
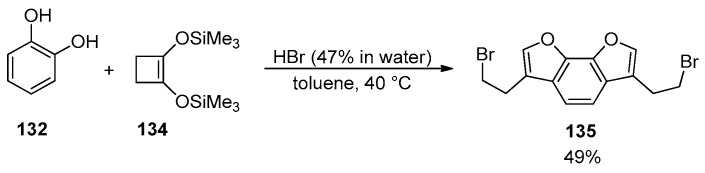
Synthesis of **BDF-2** derivative **135**.

Besides the methodologies mentioned above, it should be noted that the **BDF-2** skeleton can be synthesized by a Brønsted acid-mediated nucleophilic addition-carbocyclic rearrangement cascade reaction between catechol (**132**) with bis[(trimethylsilyl)oxy]cyclobutene (**134**) (Scheme 40) [128].

**Scheme 41:**
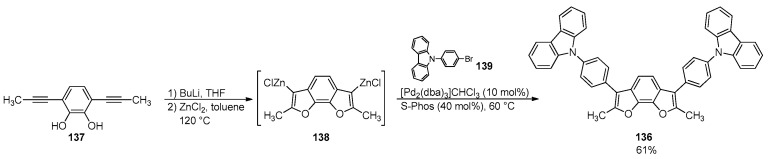
Synthesis of **BDF-2** derivative **136**.

While **BDF-2** derivative **135** was isolated in 49% yield, the same reaction using resorcinol gave a mixture of two regioisomers in 40% overall yield. In addition, substituted **BDF-2** derivative **136** can be prepared in 61% overall yield by a two-step procedure involving cyclization of 3,6-dialkynylcatechol **137** into a thermally stable organozinc intermediate **138**, followed by Negishi coupling with bromide **139** (Scheme 41) [129].

This protocol offers a divergent-oriented synthetic approach, enabling the preparation of a broad range of furan-based polyaromatic compounds [5].

**Scheme 42:**
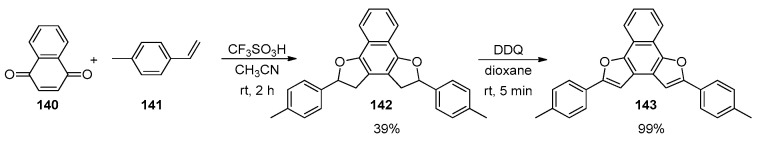
Synthesis of **BDF-1** derivative **143**.

Naphthaquinone (**140**) was also employed as a starting material for the synthesis of **BDF-1** derivatives through two different strategies, both based on a [3+2] cyclization with olefines [130] or propargyl alcohols [131]. The first approach involved a transition-metal-free oxidative C−H transformation of quinones followed by dehydrogenation. Specifically, the [3+2] cyclization of **140** with 1-methyl-4-vinylbenzene (**141**), followed by oxidative cyclization with an additional olefin molecule, afforded the tetrahydrobenzodifuran **142** (Scheme 42) [130].

**Scheme 43:**
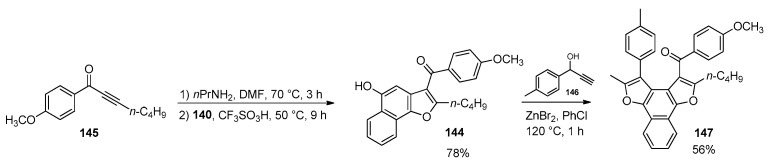
Synthesis of **BDF-1** derivative **147** via [3+2] cyclization of intermediate **144** with propargyl alcohol **146**.

Subsequent oxidation of **142** with DDQ provided the benzodifuran **143** in 39% overall yield over two steps. The second strategy involved the formation of the intermediate **144** through an aza-Michael/Michael/annulation sequence starting from ynone **145** and **140** (Scheme 43) [131].

5-Hydroxybenzofuran **144** underwent a Zn-promoted [3+2] cyclization with propargyl alcohol **146** to afford the **BDF-1** derivative **147** in 44% overall yield.

Finally, highly functionalized **BDF-1** and **BDF-2** systems were obtained by the construction of both furan rings from properly functionalized biphenols via an extended Pummerer annulation using substituted ketene dithioacetal monoxides and trifluoroacetic anhydride [132].

#### 2.2.2. Formation of a Furan Ring from Modified Benzofurans

**Scheme 44:**
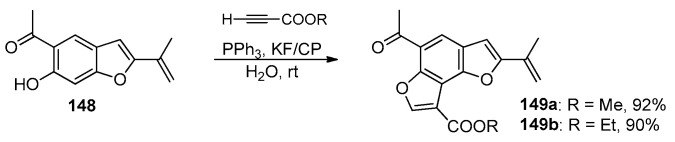
Annulation of benzofuran **148** with acetylenes for the synthesis of **BDF-3** systems **149a**,**b**.

Functionalized benzofuran **148** was successfully employed for the one-pot synthesis of **BDF-3** derivatives **149a**,**b** via a sequential addition and intramolecular cyclization reaction of **148** with terminal acetylenes in the presence of PPh_3_ and a solid base nanocatalyst made of KF impregnated on the natural zeolite clinoptilolite (KF/CP) in water at room temperature (Scheme 44) [133].

**Scheme 45:**
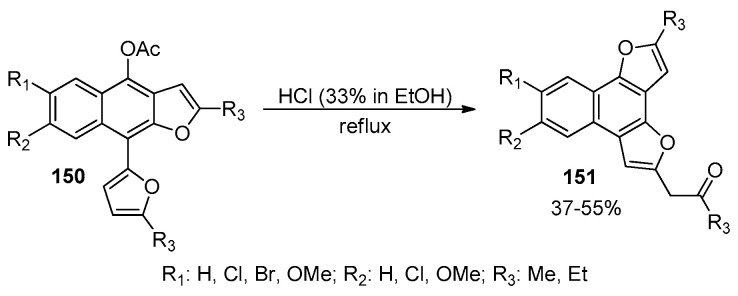
Acid-catalyzed rearrangement of naphtho[2,3-*b*]furans **150**.

Conversely, the **BDF-3** scaffold can be also formed by an acid-catalyzed rearrangement of 4-acetoxy-9-furylnaphtho[2,3-*b*]furans **150**, from which functionalized naphthodifurans **151** were isolated in 37–55% yield (Scheme 45) [134,135].

#### 2.2.3. Formation of a Benzofuran Core from Furan-Bridged Enynes

**Scheme 46:**
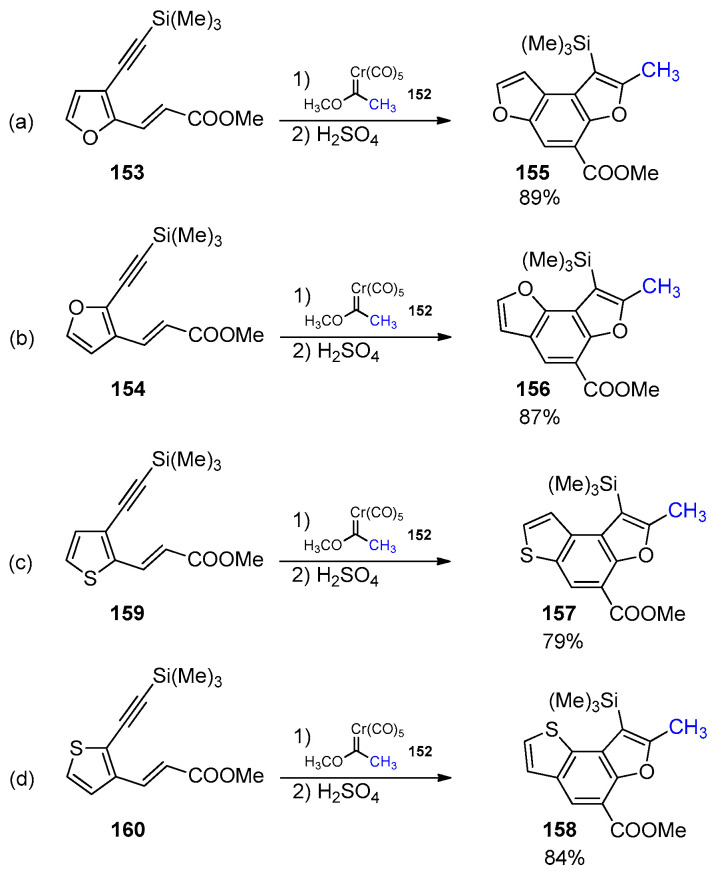
Fischer carbene coupling with enynes for the synthesis of: (**a**) **BDF-1** derivative **155**; (**b**) **BDF-3** derivative **156**; (**c**) thienobenzofuran **157**; (**d**) thienobenzofuran **158**.

A less common approach to the **BDF-1** and **BDF-3** core involved the coupling of furan-bridged enynes with carbene complexes to obtain the benzofuran core [136]. This strategy takes advantage of the well-known and robust chemistry of Fischer carbene complexes, and in this case relies on the coupling of complex **152** with conjugated dienynes **153** and **154**, in which the central alkene was included within the furan ring (Scheme 46a,b).

Both regioisomers **153** and **154** provided the corresponding **BDF-1** and **BDF-3** derivatives **155** and **156** in very good yields when reacted with **152** at 80 °C in dioxane, followed by the treatment with H_2_SO_4_ at room temperature. The same procedure was also successfully applied for the synthesis of thienobenzofurans **157** and **158**, which were isolated in 79 and 84% yield, respectively, starting from enynes **159** and **160** (Scheme 46c,d).

### 2.3. Synthesis of Benzodiselenophene (BDS) Scaffolds

**Scheme 47:**
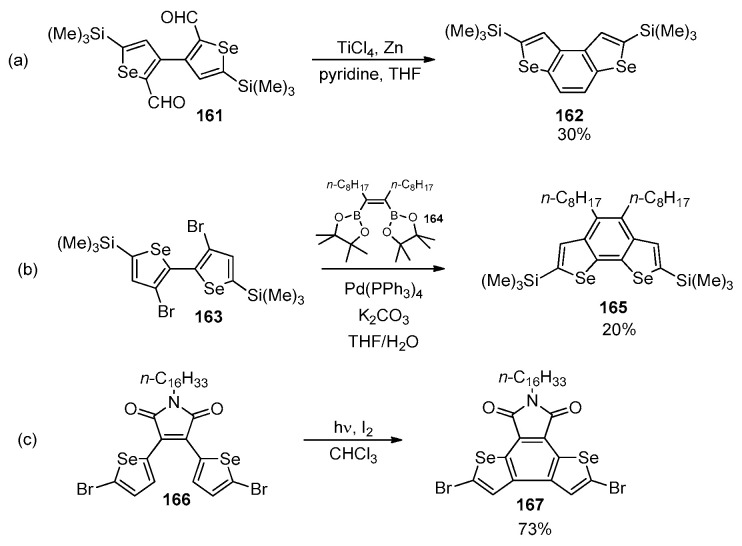
Synthesis of **BDS-1** and **BDS-2** derivatives **162** (**a**), **165** (**b**) and **167** (**c**) through the formation of the central benzene ring.

In contrast to **BDF** and **BDT** derivatives, the synthetic methodologies reported so far for the construction of benzodiselenophene core **BDS-1** and **BDS-2** are still limited. They generally involve the formation of the central benzene ring via (i) the intramolecular McMurry reaction of dialdehyde **161** to give **162** (Scheme 47a) [137], (ii) the double Suzuki coupling between functionalized 2,2′-biselenophene **163** with dioctylbis(pinacolatoboryl)alkene (**164**) for system **165** (Scheme 47b) [138], (iii) the oxidative photocyclization of dibromide **166** for compound **167** (Scheme 47c) [139].

**Scheme 48:**
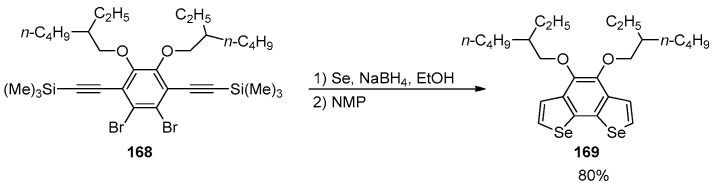
Synthesis of **BDS-2** derivative **169** through the formation of both selenophene rings.

An alternative pathway towards the **BDS-2** skeleton was also reported, and it involves the construction of both selenophene rings [140]. As reported in Scheme 48, this transformation starts with the *in situ* formation of phenylselenolate by reaction of dibromide **168** with sodium selenide, the latter being obtained by the reduction of selenium powder with NaBH_4_ in ethanol. Then, phenylselenolate species reacted with the triple bonds on the starting material, leading to the construction of both selenophene rings fused to the benzene core. The **BDS-2** system **169** was isolated in 80% overall yield.

## 3. Electrochemical Properties of BDT and BDS Derivatives and Mechanistic Insights

To comprehend the polymerization and redox behavior of **BDT** and **BDS**, one must first establish the theoretical underpinnings governing the electrochemistry of fused heterocyclic systems. The electrochemical behavior of these molecules is a direct manifestation of their electronic structure, specifically the energy and distribution of their frontier orbitals.

### 3.1. Molecular Orbital Theory: Sulfur vs. Selenium

The primary distinction between **BDT** and **BDS** lies in the heteroatom. Sulfur, a third-period element, and selenium, a fourth-period element, impart different electronic characteristics to the fused benzo-dichalcogenophene core.

#### 3.1.1. Ionization Potential and Electronegativity

Selenium is less electronegative than sulfur (2.55 vs. 2.58 on the Pauling scale) and possesses a lower ionization potential (9.75 eV for Se vs. 10.36 eV for S). In the context of a conjugated aromatic system, the heavier atom’s valence orbitals (4p for Se vs. 3p for S) are higher in energy and more diffuse. This results in a destabilization of the Highest Occupied Molecular Orbital (HOMO) in selenium-containing heterocycles relative to their sulfur counterparts. A higher HOMO energy level implies that the molecule requires less energy to remove an electron, theoretically leading to a lower electrochemical oxidation potential (*E*_ox_).

#### 3.1.2. Polarizability and Orbital Overlap

The larger size of the selenium atom enhances the polarizability of the electron cloud. This increased polarizability facilitates stronger intermolecular orbital overlap between adjacent molecules or polymer chains. In solid-state lattices, this manifests as enhanced transfer integrals, which are the quantum mechanical matrix elements describing the probability of charge hopping between sites. Consequently, **BDS** derivatives often exhibit higher charge carrier mobilities than **BDT** analogs, a trend consistently observed in OFET measurements.

#### 3.1.3. Spin-Orbit Coupling

The “heavy atom effect” introduces significant spin-orbit coupling (SOC) in selenium derivatives. While primarily relevant for photophysical transitions (promoting intersystem crossing to triplet states), SOC also influences the electrochemical stability of radical cation intermediates. The stabilization of the radical cation is crucial for the electropolymerization mechanism, as it dictates the lifetime of the reactive species and the selectivity of the coupling reaction.

### 3.2. The Electric Double Layer and Solvent Effects

Electrochemical reactions occur at the interface between the electrode and the electrolyte solution. The structure of this interface, the electric double layer, governs the kinetics of electron transfer. For hydrophobic organic molecules like **BDT** and **BDS**, the solvent choice is critical.

#### 3.2.1. Solvent–Solute Interactions

Common solvents for electropolymerization include acetonitrile (ACN), dichloromethane (DCM), and nitrobenzene. However, **BDT** and **BDS** monomers, particularly those with fused ring systems, often suffer from limited solubility in ACN. DCM is preferred for its solvency but has a narrow electrochemical window.

#### 3.2.2. Boron Trifluoride Diethyl Etherate (BFEE)

A significant advancement in the electropolymerization of thiophene-based systems is the use of boron trifluoride diethyl etherate (BFEE) as both solvent and catalyst [141]. BFEE is a Lewis acid that can form complexes with the aromatic rings, lowering the resonance energy and effectively reducing the oxidation potential of the monomer. This allows polymerization to proceed at milder potentials, avoiding the “polythiophene paradox,” where the high potential required to oxidize the monomer leads to the degradation (overoxidation) of the resulting polymer. The interaction between the Lewis acid and sulfur/selenium lone pairs stabilizes the cationic intermediates, promoting the formation of high-quality, linear polymer films with fewer structural defects.

### 3.3. Mechanisms of Electrochemical Polymerization

The formation of **BDT** and **BDS** polymers via electrochemical oxidation is a complex process involving heterogeneous electron transfer and homogeneous chemical coupling [55]. The generally accepted mechanism is the E(CE)n (Electrochemical–Chemical–Electrochemical) pathway, which proceeds through the generation of radical cations [55]. Understanding the precise sequence of bond-forming events is essential for controlling the regioregularity and molecular weight of the polymer.

#### 3.3.1. Stepwise Mechanism of Radical Cation Coupling

The polymerization initiates at the anode surface and propagates through a sequence of oxidation and coupling steps. The fundamental mechanism for **BDT** (and by extension **BDS**) can be broken down into four distinct phases: Oxidation, Dimerization, Aromatization, and Propagation.

##### Phase I: Anodic Oxidation (E)

The neutral monomer (M), diffusing from the bulk solution to the electrode surface, loses an electron to form a radical cation (M^+•^). This process is diffusion-controlled and irreversible.

M → M^+•^ + e^−^

For **BDT** and **BDS**, the highest spin density of the unpaired electron is located at the α-positions (C2 and C7) of the terminal thiophene/selenophene rings. This localization directs the regioselectivity of the subsequent coupling.

##### Phase II: Radical Copling (C)

Two radical cations couple to form a dicationic σ-dimer (M_2_^2+^). This step is the rate-determining step in the polymerization under conditions of high radical concentration (high current density) [142].

2 M^+•^⟶ (M − M)^2+^

Alternatively, a radical cation may attack a neutral monomer (M^+•^ + M ⟶ (M − M)^+•^), followed by a second oxidation. However, kinetic studies suggest the radical–radical coupling is favored due to the high local concentration of radicals at the electrode interface.

##### Phase III: Deprotonation and Re-Aromatization (C)

The formation of the σ-bond disrupts the aromaticity of the heterocyclic rings. To restore the stable aromatic system, the dicationic dimer rapidly eliminates two protons (2H^+^).

(M − M)^2+^ ⟶ M_2_ + 2H^+^

The release of protons lowers the pH at the electrode interface, which can catalyze side reactions if not managed. The resulting neutral dimer (M_2_) has a more extended conjugation length than the monomer.

##### Phase IV: Chain Propagation (E)

Because the ionization potential decreases with increasing conjugation length, the dimer (M_2_) is more easily oxidized than the monomer (*E*_ox_ (M_2_) < *E*_ox_ (M)). Therefore, at the potential applied to oxidize the monomer, the dimer is immediately oxidized to a radical cation and couples with other radicals (monomers or oligomers), extending the chain [142].

Mn^+•^ + M_m_^+•^ ⟶ M_n+m_^2+^ ⟶ M_n+m_

This cascade continues until the oligomer becomes insoluble in the electrolytic medium and precipitates onto the electrode, forming a film.

### 3.4. The Sigma σ vs. ᴨ Dimer Intermediates

Recent spectroelectrochemical and NMR studies have added nuance to the classical mechanism by identifying specific intermolecular interactions that precede bond formation [142]. This is particularly relevant for planar systems like **BDT** and **BDS**.

#### 3.4.1. The ᴨ-Dimer Assembly

Before the formation of a covalent bond, radical cations of planar conjugated systems tend to stack face-to-face to form ᴨ-dimers ([M_2_]^2+^ or [M_2_]^+•^). These aggregates are stabilized by the overlap of the ᴨ-orbitals and dispersion forces. For **BDT** and **BDS**, the large, flat aromatic core favors strong ᴨ-dimerization. This pre-organization aligns the molecules in a geometry favorable for α–α coupling, reducing the activation energy for the formation of the σ-bond.

In situ spectroelectrochemistry reveals distinctive absorption bands in the near-infrared (NIR) region attributed to these ᴨ-dimers, which are distinct from the isolated radical cation absorptions.

#### 3.4.2. The σ-Dimer Transition State

The transition from the ᴨ-stacked aggregate to the covalently bonded σ-dimer involves the rehybridization of the α-carbons from sp^2^ to sp^3^. This σ-dimer is a distinct intermediate that has been trapped and characterized in sterically hindered oligothiophenes. In **BDT** electropolymerization, bulky substituents can sterically encumber this transition, potentially slowing the polymerization rate or necessitating higher overpotentials (Table 1).

### 3.5. Regioselectivity and Structural Defects

The ideal electropolymerization of **BDT**/**BDS** occurs strictly through the α-positions to yield a linear, conjugated backbone. However, the high reactivity of the radical cation can lead to “mislinking” defects.

β-Coupling: Coupling at the β-positions disrupts the conjugation and linearity of the polymer. In fused systems like **BDT**, the β-positions are part of the fused benzene ring or adjacent to the sulfur, making them sterically and electronically less favorable for coupling than in simple thiophenes. Nevertheless, at high potentials, the selectivity decreases, leading to cross-linking and disordered films [143].

Substituent Blocking: Strategic placement of alkyl or alkoxy chains not only solubilizes the polymer but also sterically directs coupling to the α-positions, enhancing the regioregularity of the electropolymerized film [144].

### 3.6. Comparative Electrochemical Properties of BDT and BDS Monomers

The substitution of sulfur with selenium induces specific shifts in the redox properties of the monomers. These shifts are quantifiable through cyclic voltammetry (CV) and are critical for energy level matching in device applications.

#### 3.6.1. Oxidation Potentials and HOMO Levels

Experimental data from cyclic voltammetry consistently demonstrates that **BDS** derivatives are easier to oxidize than their **BDT** counterparts [145]. The oxidation potential (*E*_ox_) serves as a proxy for the HOMO energy level.

#### 3.6.2. Observed Potentials

In a direct comparison of benzo[1,2-*b*:4,5-*b*′]dichalcogenophenes, the anodic peak potential (*E*_pa_) shifts cathodically (to less positive values) as the chalcogen becomes heavier.

**BDT Monomer**: *E*_pa_ ~+0.95 V vs. Fc|Fc^+^.

**BDS Monomer**: *E*_pa_ ~+0.89 V vs. Fc|Fc^+^.

**BDTe (Tellurium) Monomer**: *E*_pa_ ~+0.48 V vs. Fc|Fc^+^.

This trend corresponds to a destabilization of the HOMO level. Using the standard approximation, $E_{HOMO}=-(E_{ox}^{onset}+4.8\mathrm{eV})$, the HOMO levels are estimated as follows:

**BDT**: −5.60 eV

**BDS**: −5.50 eV

The 0.1 eV elevation in the HOMO of **BDS** is attributed to the lower ionization potential of selenium compared to sulfur. This makes **BDS** a stronger electron donor, which is advantageous for creating low-bandgap polymers when paired with strong acceptors, but it also implies that **BDS**-based materials may be slightly more susceptible to oxidative degradation in air.

#### 3.6.3. Structural Influence on Redox Behavior

The substituents attached to the **BDT**/**BDS** core modulate these intrinsic potentials significantly (Table 2) [146].

#### 3.6.4. Alkoxy vs. Alkyl Side Chains

Alkoxy-**BDT**: The presence of oxygen atoms attached directly to the benzene core (e.g., di(alkoxy)**BDT**) raises the HOMO level significantly due to the mesomeric (+M) electron-donating effect of the oxygen lone pairs. This results in a cathodic shift of the oxidation potential by approximately 0.2–0.3 eV compared to alkyl-substituted **BDTs**. While this facilitates electropolymerization at lower potentials, it can reduce the open-circuit voltage (V_oc_) in solar cells, as Voc is proportional to the difference between the donor HOMO and acceptor LUMO.

Alkyl-**BDT**: Alkyl chains exert a weak inductive (+I) effect. Consequently, alkyl-**BDTs** have deeper (more negative) HOMO levels than alkoxy-**BDTs**. This deeper HOMO is desirable for oxidative stability and high V_oc_, but it requires higher potentials for electropolymerization, increasing the risk of overoxidation.

#### 3.6.5. Two-Dimensional Conjugation

Attaching thiophene or selenophene rings orthogonally to the main chain creates “2D-conjugated” **BDTs**.

Effect: These side groups extend the conjugation length and absorption cross-section. Electrochemically, a thienyl side group is less electron-donating than an alkoxy group. Therefore, 2D-conjugated **BDTs** typically exhibit oxidation potentials intermediate between alkyl- and alkoxy-**BDTs**, offering a balanced trade-off between stability (deep HOMO) and coverage (broad absorption).

#### 3.6.6. Band Gap Considerations

The electrochemical band gap $E_{g}^{ec}$ is defined as the difference between the onset of oxidation (HOMO) and the onset of reduction (LUMO). The substitution of S with Se consistently narrows the band gap. While the HOMO is raised, the LUMO levels of **BDT** and **BDS** are often comparable or only slightly lowered in **BDS**. The net result is a reduction in *E*_g_ by approximately 0.1 eV.

The optical band gap $E_{g}^{opt}$, determined from the absorption edge, is typically lower than the electrochemical gap due to the exciton binding energy (the energy required to separate the electron–hole pair created by photon absorption). In BDS systems, the higher polarizability of Se can reduce the exciton binding energy, bringing $E_{g}^{opt}$ and $E_{g}^{ec}$ closer together than in **BDT** systems.

### 3.7. Properties of Electropolymerized Films

While chemical coupling (e.g., Stille, Suzuki) is preferred for bulk synthesis, electropolymerization offers unique advantages for **BDT** and **BDS** derivatives [55,145,146,147]. The polymerization of **BDT** and **BDS** monomers yields conductive films whose properties are distinct from their monomeric precursors. The electropolymerization process essentially “locks” the monomer units into a conjugated backbone, creating a material with delocalized electronic states.

#### 3.7.1. Charge Carrier Mobility and Transport

One of the most compelling advantages of **BDS** derivatives is their superior charge carrier mobility. This enhancement is a direct consequence of the heavy atom effect and the structural packing it induces.

#### 3.7.2. Interchain Interaction and Transfer Integrals

The larger orbitals of selenium (4p) compared to sulfur (3p) extend further from the atomic nucleus. This extension facilitates greater orbital overlap between adjacent polymer chains in the solid state. Crystallographic and computational studies reveal that **BDS** polymers often exhibit short interchain Se•••Se distances, significantly shorter than the sum of their Van der Waals radii. These interactions create 3D networks for charge transport, allowing charge carriers (holes) to “hop” between chains more efficiently than in **BDT** polymers, where Se•••Se interactions are weaker.

In comparative studies of copolymers (e.g., with thienothiophene), **BDS**-based polymers have demonstrated hole mobilities up to an order of magnitude higher than their **BDT** analogs (1.35 × 10^−3^ cm^2^ V^−1^ s^−1^ for PBDS vs. lower values for PBDT). In highly ordered ladder-type polymers, **BDS** incorporation has pushed mobilities exceeding 0.1 cm^2^ V^−1^ s^−1^.

#### 3.7.3. Reorganization Energy

According to Marcus theory, the rate of electron transfer is inversely proportional to the reorganization energy (λ), the energy cost associated with the structural deformation of the molecule upon charging. DFT calculations indicate that **BDS** derivatives possess lower reorganization energies than **BDT** derivatives. The rigid, heavy **BDS** core undergoes less geometric distortion upon oxidation to the radical cation, thereby facilitating faster charge transfer kinetics.

#### 3.7.4. Morphology and Structural Ordering

Electrochemical growth allows for the formation of ordered films, but the degree of order depends on the monomer structure [148].

Both **BDT** and **BDS** are planar, but **BDS** polymers tend to adopt more planar backbone conformations due to non-covalent intramolecular interactions (e.g., Se•••O or Se•••F interactions with side chains or acceptors). This planarity enhances the effective conjugation length. **BDT** polymers often form amorphous or semi-crystalline films. In contrast, the strong intermolecular interactions in **BDS** polymers often drive the formation of highly crystalline domains. While this improves mobility, it can also lead to excessive aggregation, which may be detrimental for phase separation in bulk heterojunction solar cells if not carefully controlled [149].

#### 3.7.5. Redox Stability and Doping

The stability of the p-doped state (the oxidized polymer) is crucial for applications like OECTs and sensors.

PBDT: It generally exhibits excellent oxidative stability. The radical cations are stable and reversible. However, “overoxidation” at potentials > 1.2 V (vs. Ag/AgCl) can lead to nucleophilic attack by solvent impurities (e.g., water) on the backbone, breaking conjugation [150,151].

PBDS: Being more electron-rich, PBDS is easier to dope (oxidize). This allows for stable operation at lower voltages, reducing power consumption in devices. However, the higher HOMO level can make the neutral polymer prone to spontaneous oxidation by atmospheric oxygen (unintended doping), which degrades the ON/OFF ratio in transistors. To mitigate this, electron-withdrawing groups are often incorporated to deepen the HOMO while retaining the mobility benefits of the Se atom.

The comparative analysis of **BDT** and **BDS** derivatives reveals a clear dichotomy driven by the heteroatom effect. **BDT** offers a robust, chemically stable, and versatile scaffold with deep HOMO levels suitable for high-voltage applications. **BDS**, through the incorporation of selenium, unlocks superior charge transport properties, lower reorganization energies, and extended spectral response, albeit with a trade-off in oxidative stability and synthetic cost.

The mechanism of electropolymerization for these fused systems is characterized by a rapid radical cation coupling sequence, pre-organized by strong π-stacking interactions unique to their planar geometry. The identification of π-dimer intermediates highlights the supramolecular nature of the polymerization process, suggesting that controlling the aggregation state of the monomer in solution could offer a handle to tune the morphology of the resulting polymer film.

Future research will likely converge on hybrid strategies. For instance, the use of **BDT** cores with selenium-containing side chains, or random copolymers of **BDT** and **BDS**, offers a pathway to synergistic materials that combine the high voltage/stability of sulfur with the high mobility/current of selenium. Furthermore, the development of new electrolyte systems, such as ionic liquids or boron-based Lewis acids, will continue to refine the electropolymerization process, enabling the fabrication of highly ordered, defect-free films for the next generation of bio-electronics and renewable energy devices [151].

## 4. Applications

**BDT**, **BDF** and **BDS** derivatives have found widespread applications in the field of optoelectronics, especially as organic semiconductors materials for devices such as organic light-emitting diodes (OLEDs) [152], organic field-effect transistors (OFETs) [153], organic photovoltaic cells [154], and nonlinear optical materials [155]. Numerous studies have been published in recent years highlighting their relevance in these areas. Interestingly, **BDT** derivatives have also been explored in supramolecular chemistry and as chemosensors for the selective detection of toxic heavy metal ions. Moreover, although less extensively investigated compared to optoelectronics applications, **BDT** and **BDF** systems have shown potential in the biological domain.

### 4.1. Organic Light-Emitting Diodes (OLEDs)

**Figure 3:**
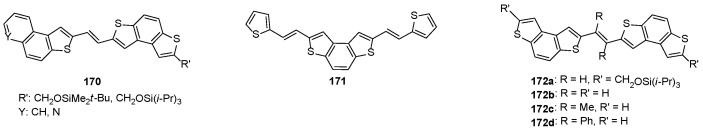
Structure of π-conjugated **BDT-1** systems **170**–**172**.

π-Conjugated polycyclic heteroaromatic compounds have been extensively studied in OLED devices, owing to their rigid π skeletons that generally confer effective luminescent properties, high carrier mobility and thermal stability. The first studies on the electroluminescence properties of **BDT-1**-based π-conjugated molecules **170**–**172** (Figure 3) were performed by K. Tanaka and co-workers starting in early 2000 [156,157].

**Figure 4:**
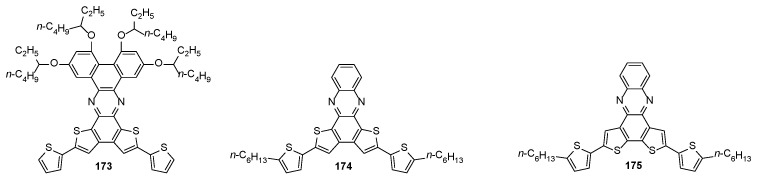
Structure of **BDT**-based emitters **173**–**175**.

The presence of a vinylene bridge between the two **BDT-1** cores was fundamental for the emission properties of compounds **172**, which were found to be highly fluorescent in solution and in thin film [156]. Different substituents on the bridging double C-C bond in **172** remarkably affected their luminescent features [156]. The flat system **172b**, in which the resonance between the two **BDT-1** cores was not perturbed, displayed quantum efficiency of fluorescence in solution (0.32) higher than those found for the twisted molecules **172c**,**d** (0.05–0.045), in which the geometrical hindrance, arising from the presence of methyl or phenyl substituents, disturbed the resonance and reduced the transition probability. A preliminary single-layered device made of ITO/**172b**/Al:Mg was prepared, and a work function value of 5.62 eV was found for **172b** by photoemission measurements [156]. More recently, OLED triplet emitters **173** and **174** (Figure 4) were reported as promising alternatives to metal–organic emitters, in which the “heavy-atom effect” hampered dissipation of electrically energy as heat [158].

Moreover, the effect of the conjugation on radiative and nonradiative relaxation of the triplet state in the two isomers **174** and **175** (Figure 4) was investigated by electroluminescence, quantum chemistry, and electron paramagnetic resonance spectroscopy [159]. The different position of the two thienyl rings significantly altered the effective conjugation path and induced different localizations of the spin density either on the phenazine unit or on the thienyl rings, while the phosphorescence was ensured by the contribution of the phenazine np* excited state. Although the thienyl rings were not necessary for generating phosphorescence, their presence increased the conjugation and then induced a red shift. The latter allowed for the development of emitters that can be used as dopants in organic semiconductor matrices.

**Figure 5:**
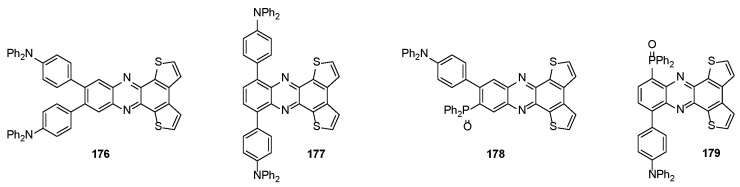
Structure of **BDT**-based TADF emitters **176**–**179**.

In 2023, red thermally activated delayed fluorescence (TADF) emitters based on decorated phenazines **176**, **177** and **178**, **179** (Figure 5), bearing a donor-acceptor-donor (D-A-D) and a donor-acceptor-acceptor (D-A-A) structure, respectively, were synthesized and their photoluminescence (PL) and electroluminescence (EL) properties were investigated [160].

The D-A-A emitters **178** and **179** displayed better PL and EL properties than those of D-A-D ones, especially in terms of the relatively higher PL quantum yields and lower nonradiative rates. An effective intra- and intermolecular charge transfer was found in **178** and **179**, due to intra- and intermolecular hydrogen bonds between the triphenylamine (D) group and the diphenylphosphine oxide (A) moiety. Moreover, the steric hindrance of the diphenylphosphine oxide efficiently inhibited concentration quenching. The device based on compound **179** exhibited the best performance (luminance max = 19,360 cd m^−2^, external quantum efficiency = 11.4% at 632 nm).

**Figure 6:**
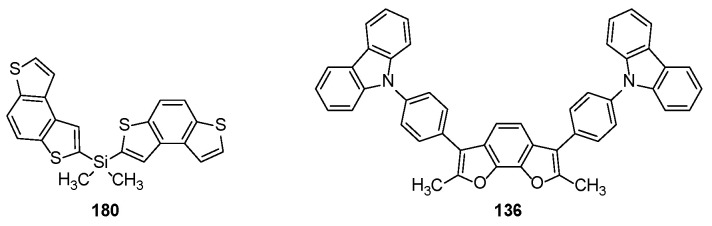
Structure of benzodithienyl silane **180** and **BDF-2**-based host material **136**.

Benzodithienyl silane **180** (Figure 6), bearing a non-conjugated 3D geometry in which a tetrahedral silicon atom links two **BDT-1** units through a dimethylsilyl bridge, was investigated as a promising semiconductive host material in devices [161].

Compound **180** crystallized into two monoclinic structures with aggregation-induced emission-like deep blue emission (390–397 nm) had a quantum yield up to 13%. Moreover, it was used as host material in green and blue emissive OLEDs to sensitize the green phosphor tris[2-phenylpyridine]iridium(III) (Ir(ppy)_3_) and the sky-blue bis[2-(4,6-difluorophenyl)pyridyl-C2,N](picolinato)iridium(III) (FIrpic), respectively. While an optimal sensitization of the green Ir(ppy)_3_ emitter was achieved, the blue FIrpic emitter was instead only partially sensitized on account of the triplets being too close. On the other hand, the **BDF-2** framework was used to develop a host material in blue phosphorescent OLED (PHOLED) devices [129]. Compound **136** (Figure 6) displayed high carrier mobilities (10^−3^ cm^2^V^−1^s^−1^) for both holes and electrons in the amorphous state, and, thanks to its high excited triplet-state energy level (*E*_T_ = 2.77 eV), it was able to sensitize FIrpic (*E*_T_ = 2.65 eV), thus demonstrating the suitability of **136** to build full-color PHOLED devices.

### 4.2. Organic Field-Effect Transistors (OFETs)

Thiophene-containing systems represent one of the most popular components used in OFETs, and numerous conjugated small organic molecules and polymers containing **BDT-1** and **BDT-2** frameworks have been synthesized and tested as semiconductors in OFETs.

#### 4.2.1. Small Organic Molecules

Several studies on the use of air-stable **BDT-1**-based π-conjugated molecule **172b** (Figure 3) as p-type semiconductors in OFETs were performed by K. Tanaka and co-workers [162,163,164,165,166]. The flat molecular plane of **172b** significantly enhances its carrier mobility due to the strong interactions between adjacent molecules. Despite this potential, these studies clearly demonstrated that the maximum hole mobility is strongly influenced by the morphology and crystallinity of the vacuum-evaporated films [167].

**Figure 7:**
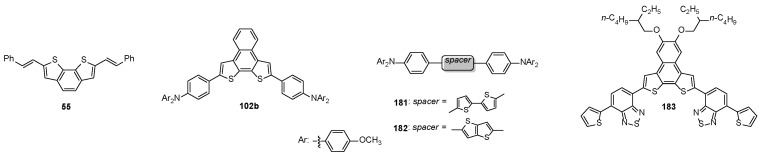
Structure of small **BDT**-based molecules used as p-type semiconductor in OFETs.

The **BDT-2**-based π-conjugated molecule **55** (Figure 7) also showed promising electrical performances in organic thin film transistors [90]. In particular, though both the **BDT-2** core and the extended π-conjugated styryl systems were not planar, the molecular arrangement in **55**-based thin films promoted an efficient charge transport across the silicon oxide semiconductor interfaces in an organic thin film transistor configuration. The dithienonaphthalene **102b** (Figure 7) also worked as p-type semiconductor in OFET [116], with better performance than those of thiophene- and thienothiophene-based derivatives **181** and **182** (Figure 7) [168].

Alternatively, the push–pull semiconductor system **183** (Figure 7), containing the **BDT-2** scaffold as donor alternated to the benzothiodiazole core as acceptor, displayed hole mobility (1.4 × 10^−2^ cm^2^ V^−1^ s^−1^) in OFET devices [169].

**Figure 8:**
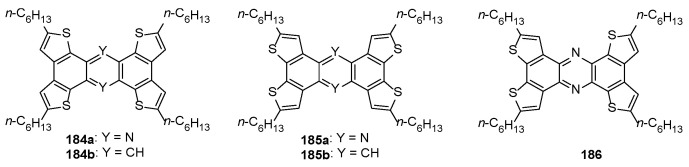
Structure of phenazines **184a**, **185a**, **186** and anthracene analogs **184b** and **185b**.

Numerous π-extended polycyclic aromatic hydrocarbon systems incorporating the **BDT-1** core were found to be stable and promising low-molecular weight organic semiconductors in OFETs [41,52,68,71,72,73]. OFET devices of alkylated phenazines **184a**, **185a** and **186** incorporating **BDT-1** and/or **BDT-2** cores were fabricated by vacuum deposition, and their performances were compared with those of anthracene analogs **184b** and **185b** (Figure 8) [170].

**Figure 9:**
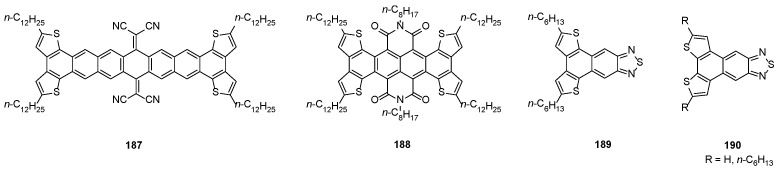
Structure of **BDT**-based ambipolar charge transport compounds **187**–**190**.

Thin films of **184a**, **185a** and **186** showed nearly the same crystallinity and then similar FET features (around 10^−6^ cm^2^ V^−1^ s^−1^ when deposited at 75 °C), regardless of the position of sulfur atoms. On the other hand, lower FET mobilities were obtained for **BDT** systems than those of anthracene analogs **184b** and **185b** (around 10^−2^ cm^2^ V^−1^ s^−1^) [58,68], presumably due to lower donor abilities and the larger reorganization energies of the nitrogen-containing systems. **BDT-1** and **BDT-2**-based semiconductors with ambipolar charge transport properties were also synthesized, and their electrical behavior was examined [60,74,78]. The ambipolar charge transport in field-effect transistor devices was observed for the fused heteroaromatic compounds incorporating donor/acceptor structures, including molecule **187** [78], **188** [74] and fused dithieneonaphthothiadiazoles **189** and **190** [60] (Figure 9).

#### 4.2.2. Polymers

**Figure 10:**
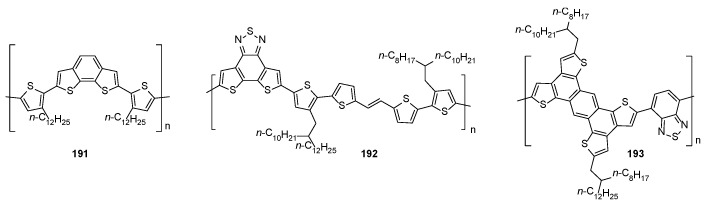
Structure of polymers **191**–**193** used in OFETs.

Besides small organic molecules, π-conjugated semiconducting polymers containing the **BDT-1** and **BDT-2** framework were found to be promising high-performance OFETs. The introduction of the **BDT-2** core into a semiconducting polymer backbone **191** (Figure 10) led to an active material in OFET [171]. This polymer displayed high charge-carrier mobility (0.5 cm^2^ V^−1^ s^−1^) and was found to be suitable for application on flexible substrates (i.e., PET film). The curvature of the **BDT-2** core ensured the best compromise between solubility and aggregation tendency towards the quick formation of highly ordered films.

An alternative approach towards semiconducting polymers for use in high-performance OFETs relies on the copolymerization of donor and acceptor units to yield D/A copolymers with high charge-carrier mobility. **BDT** frameworks were found in π-extended systems which were employed as building blocks to create alternating D/A copolymers such as **192** [172] and **193** [173] (Figure 10). In these systems, the strong D-A interactions in combination with the highly coplanar polymer backbone guaranteed π–π stacking self-assembly and a compact solid-state packing associated with high charge-carrier mobility in OFETs, while the presence of long branched alkyl chains (e.g., 2-octyldodecyl) improved the solubility in organic solvents.

**Figure 11:**
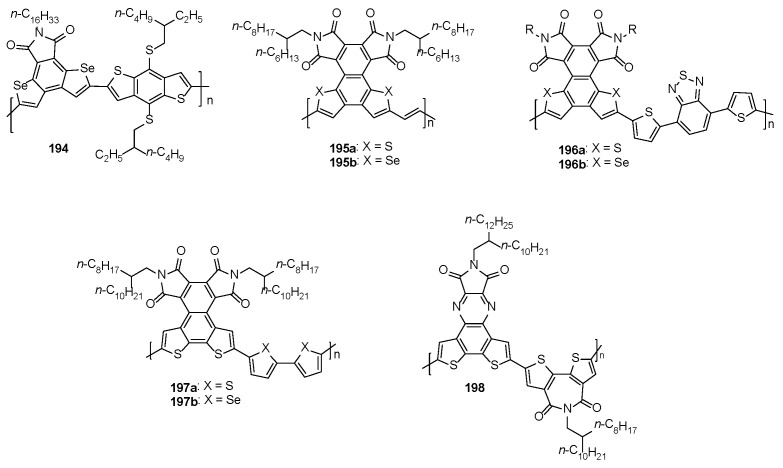
Structure of polymers **194**–**198**.

π-Conjugated D/A polymers containing **BDT** and **BDS** cores incorporated into one or two-electron-poor imide units, such as **194** [139], **195** [174], **196** [174] and **197** [175] (Figure 11), were also tested as semiconductors in OFET devices.

The nature of chalcogen atoms in combination with the design of the copolymers **195** and **196** significantly affected their charge transport properties. The average/maximum electron mobility of **BDS**-based polymer **195b** (0.005/0.01 cm^2^ V^−1^ s^−1^) were an order of magnitude higher than those of **BDT**-based polymer **195a** (4.2 × 10^−4^/7.1 × 10^−4^ cm^2^ V^−1^ s^−1^). Conversely, the average/maximum electron mobility of **BDT**-based copolymer **196a** (0.003/0.005 cm^2^ V^−1^ s^−1^) was an order of magnitude higher than that of **BDS**-based polymer **196b** (3.5 × 10^−4^/5 × 10^−4^ cm^2^ V^−1^ s^−1^). Overall, the electron mobility of most of these systems is comparable to the classic n-type semiconductor (i.e., PC_71_MB ca. 10^−3^ cm^2^ V^−1^ s^−1^) [176]. The polymer **198** (Figure 11), containing a strong electron-deficient dithiene-fused quinoxalineimide, displayed unipolar n-type transport character with an electron mobility of 0.25 cm^2^ V^−1^ s^−1^ in OFETs [177].

### 4.3. Organic Solar Cells (OSCs)

Functionalized **BDT** systems along with **BDT**- and **BDS**-based polymers have been found to be promising organic semiconductors for the development of bulk heterojunction (BHJ) OSCs, dye-sensitized solar cells (DSSCs), and perovskite solar cells (PSCs).

#### 4.3.1. Bulk Heterojunction (BHJ) OSCs

BHJ solar cells, reported for the first time by Yu and co-workers in 1995 [178], are still one of the most promising OSCs in photovoltaic technology [179]. They are made of an active layer formed by a conjugated organic small molecule or a polymer as a donor (p-type semiconductors) and a fullerene derivative or a non-fullerene small molecule or polymer as an acceptor (n-type semiconductor). **BDT-1** and especially **BDT-2** frameworks were used in the form of small molecules or polymer species, either as p-type or n-type semiconductors in BHJ OSCs. More recently, a few examples of **BDS-2**-based polymers as donors in BHJ OSCs were also reported.

##### Polymer Donors Based on **BDT** and **BDS**

**Figure 12:**
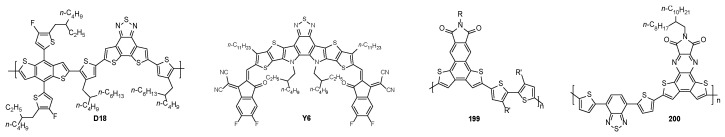
Structure of **D18**, **Y6** and **BDT**-based polymers **199** and **200** used as donors in BHJ cells.

The donor–acceptor (D-A) copolymer **D18** (Figure 12), which alternates the electron-donating (D) benzo[1,2-*b*:4,5-*b*′]dithiophene and the electron-accepting (A) **BDT-2** fused-ring benzothiadiazole, represents one of the most interesting high-performance donor polymers in BHJ systems [180]. **D18** was proposed for the first time by Ding and co-workers [181] and, when it was blended with the small acceptor molecule **Y6** (Figure 12), a remarkable power conversion efficiency (PCE) up to 18.22% was achieved. More recently, thanks to their high device performances and suitable morphological characteristics, polymer donors based on **D18** and its analogs were also successfully applied in all-polymer solar cells, obtaining PCE up to 19% [182,183,184].

Fullerene-based BHJ solar cells were also fabricated using donor copolymers containing the **BDT-1** framework, such as the A-D structures **199** (Figure 12) [76], made of **BDT-1** containing imide (A) and 2,2′-bithiophene units (D), and the structure D-A_1_-D-A_2_ **200** (Figure 12), composed by the benzothiadiazole acceptor (A_1_), the **BDT-1** fused-system (A_2_) and the thiophene ring as donor (D) [75]. However, in both cases, modest PCEs were obtained (2.45–6.21%).

**Figure 13:**
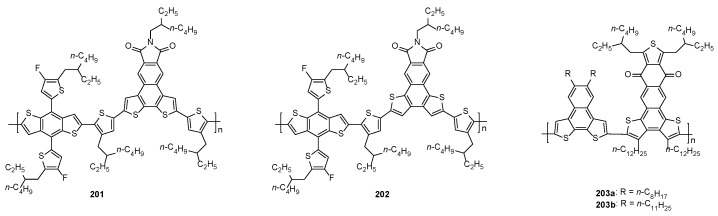
Structure of **BDT**-based copolymers **201**–**203**.

In 2022, a comparative study demonstrated that the orientation of the two thiophene rings in the acceptor unit of donor copolymers **201** and **202** (Figure 13) significantly affected their spectral and morphological properties as well as their efficiency in BHJ devices using **Y6** as the acceptor [102]. Indeed, while the device fabricated with the **BDT-2**-based copolymer **201** provided a PCE of 15.05%, the use of the **BDT-1**-based copolymer **202** afforded devices with almost no solar cell performance.

On the other hand, wide-bandgap D-A donor copolymers **203a**,**b** (Figure 13) were employed to develop BHJ solar cells blended with **Y6** as a non-fullerene acceptor [185]. In this case, the **BDT-2** scaffold represented the donor unit (D) while the **BDT-1** portion was the acceptor one (A), and a PCE up to 16.19% was achieved in the ternary devices made of a mixture of **203a**:**203b** blended with **Y6**.

Examples of **BDT-1** [186], **BDT-2** [104,187,188] and **BDS-2** [140,189] cores used as donor moiety in the donor-acceptor (D-A) copolymers were also reported as donor polymers in BHJ systems, though low to moderate PCE values were achieved (up to 11%).

##### Acceptors Based on **BDT**

**Figure 14:**
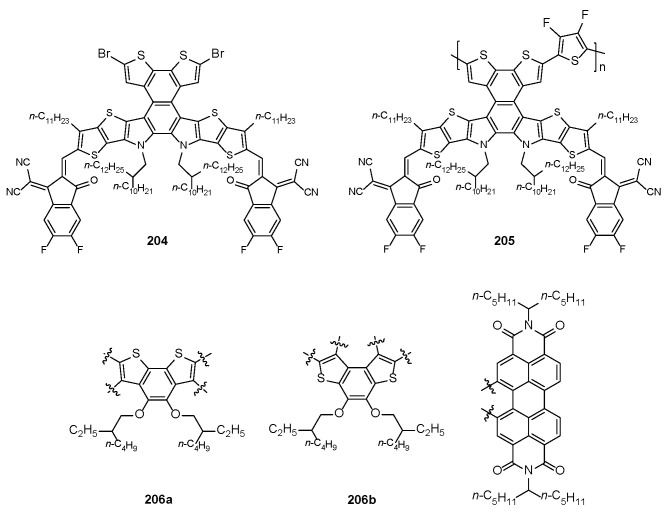
Structure of **BDT**-based acceptors **204**–**206** in BHJ solar cells.

As far as the use of **BDT**-based systems as n-type semiconductors in BHJ solar cells, non-fullerene small-molecule-acceptor **204** [190] and the acceptor polymer species **205** [191], (Figure 14), both incorporating the **BDT-2** on the quinoxaline-fused core were used to develop OSCs with PCE ranging from 14.14 to 17.05%.

Interestingly, when the two isomeric **BDT-2** and **BDT-1** unit were fused at the bay position of perylenediimide skeleton, the corresponding n-type organic semiconductors **206a**,**b** (Figure 14) displayed different device performances in non-fullerene OSCs, and in this case the higher PCE value was also achieved by the **BDT-2**-based acceptor **206a** (4.44%) in comparison with that provided by the **BDT-1** system **206b** (2.98%) [192].

#### 4.3.2. Dye-Sensitized Solar Cells (DSSCs)

**Figure 15:**
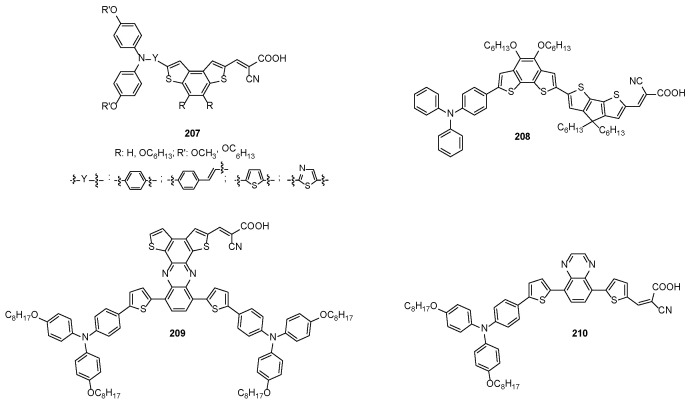
Structure of **BDT** containing push–pull systems **207**–**210** for DSSCs.

After the study reported by Gratzel and O′regan in 1991 [193], DSSCs gained a lot of attention as a low-cost and renewable energy source in photovoltaic technology [194]. The dye sensitizer represents the key element of a DSSC, and it is responsible for the light-harvesting and charge-separation processes. Organic dye is generally composed of an electron donor unit (e.g., alkoxyl substituted triarylamines) and an electron acceptor unit (e.g., cyanoacrylic acid), which are covalently connected through a π-conjugated spacer, whose structure significantly affects the photophysical properties of the dye. In 2012 [195] and 2013 [196], the **BDT-1** framework was used as a π-spacer in dye sensitizers **207** (Figure 15), and power conversion efficiencies (η) ranging from 3.6 to 5.6% in a liquid cell were achieved. These similar values indicate that the nature of the heteroaromatic linkers (i.e., benzene, thiophene and thiazole) does not have an appreciable influence on efficiency. In 2017, the **BDT-2** core was employed as a π-spacer to yield sensitizers **208** (Figure 15), in which a heteroaromatic fragment between **BDT-2** and the acceptor unit was inserted to improve the light harvesting of the dyes. PCE values higher than 9% were obtained using the I^−^/I^3−^ or Co(phen)_3_^2+/3+^ as an electrolyte in the presence of chenodeoxycholic acid as co-adsorbent [197].

The near-infrared co-sensitizer **209** (Figure 15), containing the electron-withdrawing **BDT-1** framework, and the co-sensitizer **210** (Figure 15) were mixed to set quasi-solid-state DSSCs, which exhibited PCE up to 8.04% and long-term stability (1000 h under continuous light soaking) [198].

**Figure 16:**
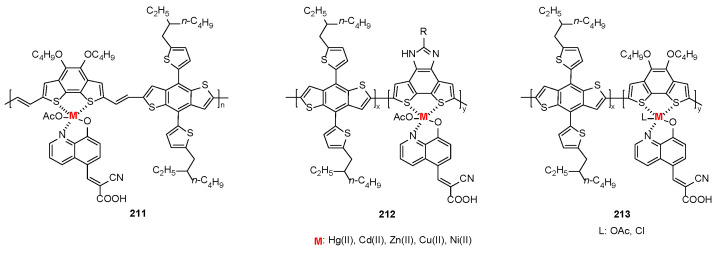
Structure of metal-based polymeric sensitizers **211**–**213**.

More recently, Zhong and co-workers developed a family of metal complexes containing the sulfur coordination of the **BDT-2** core, which were used as monomers to create dye sensitizer copolymers for DSSCs [199,200,201]. These metal-based polymeric sensitizers **211**–**213** (Figure 16) are characterized by a D-A1-π-A2 motif, in which benzo[1,2-*b*:4,5-*b*′]dithiophene cores were used as electron donors (D), the metal complexes containing the **BDT-2** ligand acted as auxiliary electron acceptors (A1), and 8-quinolinol derivatives were used as π-bridge and electron acceptor (A2) units.

Five metals (i.e., Ni, Cu, Zn, Cd and Hg) were selected to synthesize the corresponding polymers and to study their performance in DSSC devices. As a general trend, PCEs increased with the increasing radii of Ni(II), Cu(II), Zn(II), Cd(II) and Hg(II), and the best photovoltaic performance was achieved with Hg(II)-based polymers (10.96 [199], 11.78 [200] and 12.89% [201]).

Although the metal ions displayed the same charge, their increasing ionic radius strengthened the coordination with the sulfur of **BDT-2**. This, in turn, enhanced both the electron-withdrawing capability and the intramolecular electron transfer of the metal-based acceptors A1. This stronger interaction improved the electron absorption capacity and the push–pull electron balance, narrowed the polymer bandgap, induced the redshift of λ_max_, and boosted photovoltaic performance.

#### 4.3.3. Perovskite Solar Cells (PSCs)

**Figure 17:**
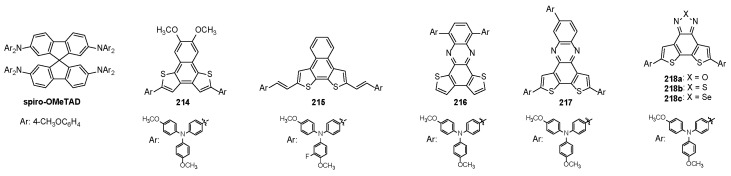
Structure of **spiro-OMeTAD** and **BDT**-based HTMs **214**–**218** employed in PSCs.

Perovskite solar cells (PSCs) represent younger devices than BHJ OCs and DSSCs, since the first example of PSCs was reported in 2009 by Miyasaka and co-workers, in which a PCE of 3.8% was obtained [202]. Since this work, PSCs have rapidly attracted much attention due to their impressive capability of converting solar energy into electricity with skyrocketing PCEs, currently exceeding 25%. Generally, PSCs consist of five parts, including a transparent conductive metal electrode, a hole-transporting material (HTM), a perovskite light absorber layer, an electron transport layer, and a black electrode of carbon or noble metal. The HTM collects and transports photo-generated holes between the absorption layer and electrode of PSCs, realizing effective separation of electrons and holes, which is an essential point to enhance PCE of PSCs [203]. Organic HTMs based on conductive small molecules or polymers have been widely investigated in high-performance PSCs, and the **spiro-OMeTAD** (Figure 17) still represents one of the most advantageous HTMs in PSCS technology, though some obstacles towards large-scale applications have led to further exploration of alternative and efficient HTMs.

Small-molecule semiconductors containing the **BDT-1** and **BDT-2** frameworks were employed as HTMs in PSCs, demonstrating great potential thanks to their high hole mobility, thermal stability, and tunable molecular structures. Naphto **BDT-1** [64,204] and **BDT-2** [116] were incorporated as π-bridges to connect the two electron-rich triphenylamine (TPA) units in HTMs **214** (Figure 17) and **102b** (Figure 7), respectively. For compound **214**, the PCE reached considerable values up to 18.8%, slightly higher than the **spiro-OMeTAD** (18.1%). More recently, HTM **215** (Figure 17), bearing a carbon-carbon double bond as π-spacer between the **BDT-2** core and the triarylamine units, showed very good PCEs up to 17.2% [205], without any device oxidation treatments, which are generally required for **spiro-OMeTAD**.

Alternatively, π-bridges made of **BDT-1** [206] and **BDT-2** [207] incorporated into a quinoxaline core provided D-A-D-type HTMs **216** and **217** (Figure 17), respectively, with the TPA as donors (D) and the quinoxaline derivatives as acceptor units. These HTMs displayed photovoltaic performances competitive with **spiro-OMeTAD**, and PCEs over 20% with excellent long-term stability were achieved by PSC devices using the dopant-free **216** (21.03%) and the doped HTM **217** (20.52%).

Finally, three D-A-D dopant-free HTMS **218** (Figure 17) were designed and tested in PSCs, in which two TPA donor units were linked to different acceptors made of fused **BDT-2** chalcogenadiazoles (i.e., furazane, thiadiazole and selenadiazole) [208]. The nature of the chalcogen atom in the diazole framework had a remarkable effect on the optical, electrochemical, and charge-transport features of **218**, and the best photovoltaic performance was obtained in dopant-free PSCs made of selenium-containing HTM **218c** (PCE for **218c** of 15.09% vs. 13.31% for oxygen derivative **218a** and 11.65% for sulfur derivative **218b**).

### 4.4. Nonlinear Optical (NLO) Materials

Nonlinear optical (NLO) compounds generally include donor and acceptor groups linked through a π-conjugated spacer, and, among them, organometallic species exhibit interesting NLO properties thanks to the coordination of the metal atom to conjugated ligands that promote effective charge transfer transitions [209]. The **BDT-1** framework was used as a ligand for coordination to the metal center of [Fe_2_(η^5^-C_5_H_5_)_2_(CO)_2_(μ-CO)(μ-C–CH_3_)]^+^[BF_4_]^−^ [210] and a family of cyclopentadienyl iron/ruthenium derivatives [211].

**Figure 18:**
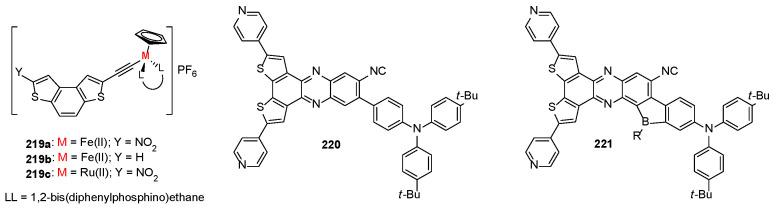
Structure of **BDT-1**-based metal complexes **219** and **BDT-2**-based donor–acceptor systems **220** and **221**.

First studies on the quadratic hyperpolarizabilities (*β*) of iron and ruthenium complexes **219** (Figure 18) were performed by hyper-Rayleigh scattering measurements at 1500 nm, obtaining a value of *β*_0_ = 19 × 10^−30^ esu for **219a**, and irrelevant values for **219b**,**c** [211].

Besides organometallic complexes, the development of organic molecules with donor–acceptor architecture and a π-conjugated bridge also represents an effective approach towards second-and/or third-order NLO materials with enhanced optical hyperpolarizabilities [212]. Two intramolecular charge-transfer (ICT) compounds **117a**,**b** (see Scheme 36), in which the cyano groups acted as acceptors while the angular-shaped structure of the π-conjugated **BDF-2**-based bridge provided efficient ICT processes, exhibited low-dimensional microstructures with significantly distinctive linear and nonlinear optical properties, as a consequence of diverse orientations of their transition dipoles and self-assembled structures [122]. More recently, the intramolecular boron-locking strategy was employed in a series of **BDT-2**-based donor-acceptor compounds, such as **220** and **221** (Figure 18), to evaluate how torsion angles θ_1_ and θ_2_ between the donor unit (i.e., the triphenylamine) and the boron-locking acceptor affected the first hyperpolarizability (*β*) value [213]. In particular, the decrease in the torsion angles in **221** significantly increased the *β* value by up to 94%, likely due to the lower excited energy of the key excited state and enhanced charge transfer from the triphenylamine group to the **BDT**-pyridine moiety.

### 4.5. Self-Assembly

Shape-persistent macrocycles (SPMs) are promising compounds in the field of supramolecular chemistry and self-assembly because they present an inner cavity in the nanometer regime, and the building blocks of the ring are rather rigid and connected in such a way that the whole structure cannot collapse. Due to their rigidity, SPMs can be functionalized independently in their interior and exterior, and the orientation of the side groups remarkably influences the properties and applications of SPMs [214]. SPMs containing thiophene-based units are interesting systems to build self-assembled monolayers at the solid/liquid interface and for application in photovoltaics. The most significant contribution in this field was provided by S. Höger and co-workers, who developed different families of π-expanded shape-persistent macrocycles containing **BDT-1** [215,216,217] and **BDT-2** [218,219,220] units.

**Figure 19:**
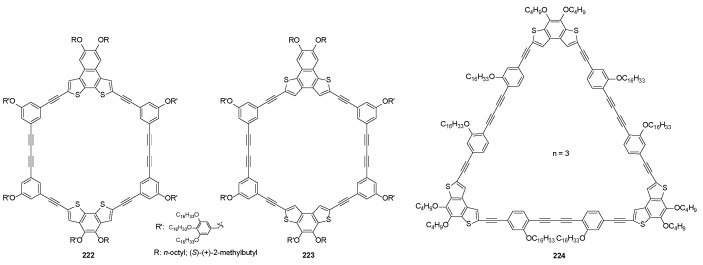
Structure of macrocycles **222**–**224**.

Macrocycles **222** and **223** (Figure 19), containing phenylene-ethynylene-butadiynylene backbone alternated to naphtho[2,1-*b*:3,4-*b*′]dithiophene [218] and benzo[1,2-*b*:4,3-*b*′]dithiophene [216] units, respectively, yielded 2D ordered arrays on highly oriented pyrolytic graphite (HOPG) by self-assembly under ambient conditions. The macrocycle arrays ordered from **222** represented a suitable template for the epitaxial co-adsorption of metallacycles, thanks to the presence of electron rich **BDT-2** units that favored the electronic interactions with the electron withdrawing guest molecules, while macrocycles **223** afforded supramolecular empty helical nanotubes in the liquid crystalline mesophases.

**Figure 20:**
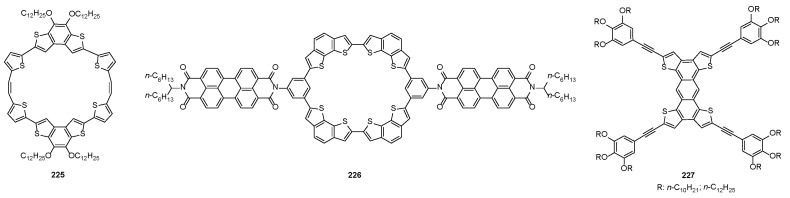
Structure of macrocycles **225**–**227**.

Höger’s group also reported the synthesis of SPMs [224]_n_ (n = 3–6) (Figure 19) based on **BDT-1** corner pieces connected through phenylene-ethynylene-butadiynylene units, in which the ring sizes and the extra-annular alkoxyl chains ensured enough solubility and elastic deformability of the systems [215]. All macrocyclic oligomers, when adsorbed under ambient conditions at the interface of diluted solutions of [224]_n_ in 1,2,4-trichlorobenzene (TCB) as solvent (10^−4^–10^−7^ M) and HOPG as substrate, assemble to highly ordered 2D patterns, supporting the formation of molecular porous and dense long-range ordered patterns of complementary shapes. Thereafter, A. Bedi and S. Zedi synthesized a macrocycle **225** (Figure 20), containing two **BDT-1** units linked through thienyl ethylene spacers [217]. This macrocycle underwent self-assembly in the solid state, forming microfibers of the length of several μm and thickness of ∼400 nm on the Si/SiO_2_ surface. The SPM donor-acceptor hybrid system **226** (Figure 20) was also synthesized [219], in which the electron rich macrocycle backbone, made of four naphto[1,2-*b*:4,3-*b*′]dithiophene units connected through two phenylene rings, was decorated with two extra-annular electron poor perylene bisimide groups. This system formed supramolecular 1D aggregates at the TCB/HOPG interface, mainly governed by the strong attractive forces between the acceptor perylene bisimide moieties. Moreover, a deep study of its charge separation properties through different electron paramagnetic resonance (EPR) techniques in combination with light excitation showed the presence of intermolecular and intramolecular charge separation, and the observed radical pair states induced upon illumination made hybrid systems like **226** as promising semiconductors in organic photovoltaic cells [220].

More recently, **BDT-1** based tetrathienoanthracenes **227** (Figure 20) were synthesized and used as planar discotic fragments for the development of π-conjugated discotic liquid crystal (DLC) systems as hole-transporting materials bearing a supramolecular columnar architecture [221]. The great tendency of **227** to align homeotropically in the columnar mesophase over a large area in space-charge limited current (SCLC) cells in combination with the strong co-facial π–π interactions and multiple S^…^S contacts between the tetrathienoanthracene core ensured a remarkably high hole mobility (4.22 cm^2^V^−1^s^−1^) for this DLC system in SCLC device.

### 4.6. Chemosensors

Methods based on fluorescent and colorimetric chemo-sensory materials for the selective recognition of biologically and environmentally toxic heavy metal ions (e.g., Hg^2+^, Pb^2+^) has been receiving much attention due to their excellent sensitivity and selectivity, along with their response time, local observation, nondestructive character, economic nature, and synthetic simplicity.

**Figure 21:**
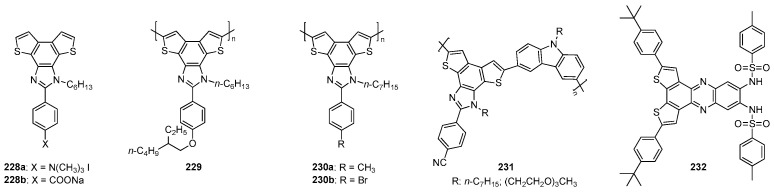
Structure of **BDT** derivatives **228**–**232** employed as chemosensors.

In particular, the development of highly sensitive chemosensors with fluorescence turn-off or turn-on sensing mechanism for the specific recognition of toxic metal ions analyte represents an important objective in chemistry, biology and medicine. **BDT-1**-based small molecular organic and π-conjugated polymeric chemosensors **228** and **229** (Figure 21), respectively, with neighboring nitrogen and sulfur heteroatoms as chelating sites for the selective detection of bivalent cations were reported [222,223]. Organic molecules **228** displayed remarkable sensitivity towards Pb^2+^ over the other metal ions in aqueous solutions [222], while π-conjugated polymer **229** was found to be a suitable system for both colorimetric and ratiometric detection of Hg^2+^ and the fluorometric detection of Zn^2+^ through a fluorescence turn-on response with an enhanced fluorescence lifetime in the presence of Zn^2+^ [223]. Moreover, the **229**-Zn complex allowed the selective colorimetric detection of I^−^ over other anions.

Similar polymers **230** (Figure 21) acted as fluorescence sensors for the selective sensing of biological and environmentally relevant Cu^2+^ [224], since the presence of both hard N and soft S donors made **230** efficient fluorescence sensors towards slightly acid transition metal cations such as Cu^2+^. Polymer **230b** exhibited outstanding sensitivity and selectivity towards Cu^2+^ by emission quenching via photoinduced electron transfer, and its polymeric film was also investigated as a thin-film polymeric sensor for application in on-site detection of Cu^2+^. In this case, the sensing ability of these polymers was strictly dependent on the electronic nature of the substituents on the phenyl ring of **230**, since **230b**, with a *p*-bromophenyl pendant, succeeded over the toluene-based congener **230a** towards Cu^2+^ sensing. Emissive and well-defined π-conjugated copolymers **231** (Figure 21) were found to be low-cost and highly selective and sensitive turn-off fluorescent probes for the selective detection of Hg^2+^, with a limit of detection up to ppb level (40–50 ppb) in semi-aqueous environment [225]. The study of optical probes for the qualitative or quantitative detection of anions also represents an important target for health and environment. The anion-responsive **BDT-2**-based molecule **232** (Figure 21) exhibited especially strong interaction with carboxylate, dihydrogen phosphate and cyanide anions, through H-bonding with the two N−H bonds of sulfonamide groups [226].

### 4.7. Biology

Although **BDT** and **BDF** frameworks have been extensively studied for their optoelectronic properties in materials science, their application in biological domain, while less common, has also been documented.

**Figure 22:**
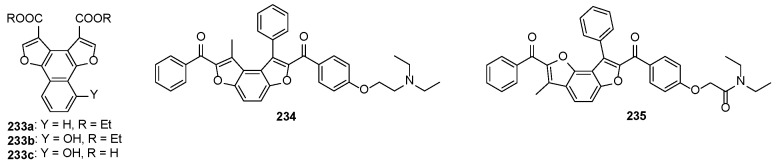
Structure of biologically relevant **BDF** derivatives **233**–**235**.

Some **BDF-1** and **BDF-2** derivatives displayed cytotoxic activity against different human cell lines. The in vitro cytotoxicity of **BDF-1** systems **233** (Figure 22) against human hepatocellular liver carcinoma cell line (HepG2) and their antioxidant activity using 2,2-diphenyl-1-picrylhydrazyl (DPPH) and 2,2′-azino-bis-3-ethylbenzthiazoline-6-sulphonic acid (ABTS) bioassays were evaluated and compared with those of three naturally occurring naphthoquinones (i.e., juglone, lawsone, and plumbagin) [227].

Compounds **233b**,**c** showed similar and higher scavenging activity in DPPH assay in comparison with that of natural naphthoquinones, while comparable activity for all molecules was observed in ABTS bioassay. Conversely, no cytotoxic activity was observed against HepG2 cell line for derivatives **233b**,**c** at all concentrations (up to 100 mg/mL), but a significant anti-proliferative activity was revealed for **BDF-1 233a** (IC_50_ 26.05 mg/mL), higher than juglone (IC_50_ 48.61 mg/mL) and plumbagin (IC_50_ 58.63 mg/mL).

A set of **BDF-1** and **BDF-3** derivatives were tested in vitro for the inhibition of human breast cancer cell lines T-47D and MCF-7 [228]. Compounds **234** and **235** (Figure 22) were found to be the most promising selective estrogen receptor modulators (SERMs) with IC_50_ values similar to that of the tamoxifen.

**Figure 23:**
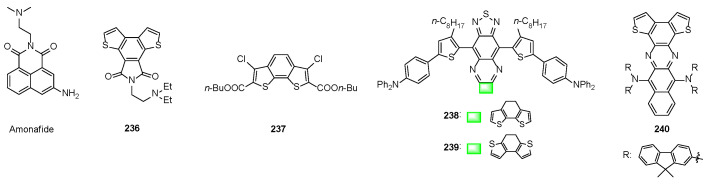
Structure of Amonafide and **BDT** derivatives **236**–**240** employed in biological studies.

**BDT** derivatives were also designed and applied in biological field, demonstrating potential both as anticancer agents and as fluorophores for bioimaging applications. The antiproliferative activity of **BDT-1** derivative **236** (Figure 23) in cultured rat aortic smooth muscle cells (SMCs) and in the human NCI-H460 lung tumor cell line was comparable to that achieved by Amonafide (Figure 23) that was included as a reference compound [229]. DNA intercalating assay demonstrated that **236** did not directly interact with DNA, so its antiproliferative effect was not a consequence of its DNA intercalating activity.

The inhibitory activities of various **BDT-2** systems against alpha-glucosidase, urease, and the free radical production were evaluated [230]. Among them, compound **237** (Figure 23) displayed significant DPPH radical scavenging activity and exhibited promising alpha-glucosidase inhibition relative to other tested molecules. Structure-activity relationship studies suggested that the presence of the two chlorine atoms play a key role in its activity.

The incorporation of a **BDT-2** and a **BDT-1** framework onto the structures **238** and **239** (Figure 23), respectively, enabled the development of novel fluorophores emitting in the second near-infrared (NIR-II) window for biomedical applications [231]. Notably, **239**-based nanoparticles exhibited bright NIR-II emission, allowing in vivo imaging with high signal-to-background ratios and facilitating long-term stem cell tracking for acute lung injury detection. Compound **240** also displayed an absolute QY of 0.4% with extension to 1400 nm, making it highly suitable for NIR-II bioimaging [232]. Furthermore, cerebrovascular function, including cerebral blood flow and vascular reactivity under various conditions, was accurately quantified.

**Figure 24:**
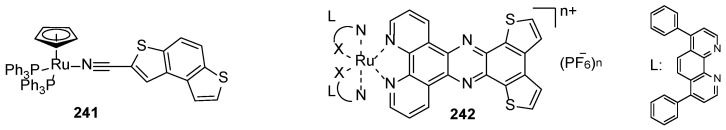
**BDT-1**-based Ru(II) complexes **241** and **242**.

**BDT-1** was also employed as a ligand in cationic Ru(II) complex **241** (Figure 24), which showed higher cytotoxicity than cisplatin against human leukemia cancer cells (HL-60 cells) [233]. This activity could be ascribed to some DNA interactions as evidenced by CD, electrophoretic mobility and AFM studies. The most plausible interactions rely on weak ligand–DNA interactions through hydrogen bonds.

More recently, incorporation of a **BDT-1** framework into a classic DNA–intercalative Ru(II) complex with chemotherapeutic activity yielded a two-photon absorption Ru(II) complex **242** (Figure 24) [234]. This complex is localized to both mitochondria and nuclei, enabling the regulation of DNA-related chemotherapy mechanisms such as DNA topoisomerase and RNA polymerase inhibition. It demonstrated remarkable phototherapeutic efficacy with minimal toxicity to normal liver and kidney cells in vitro, and in vivo it showed high antitumor activity against malignant melanoma and cisplatin-resistant NSCLC, achieving 100% mouse survival, low toxicity to normal cells, and minimal residual tumor rates.

### 4.8. Miscellaneous

#### 4.8.1. BDT-Based Porous Organic Polymers (PSCs)

**Figure 25:**
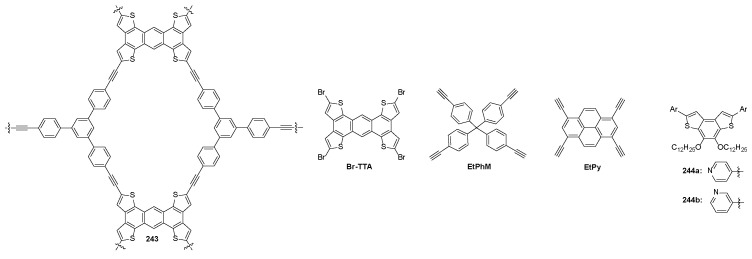
Structure of: **BDT-1**-based CMP **243**, **Br-TTA**, **EtPhM**, **EtPy**, and multifunctional **BDT-1** derivatives **244**.

Porous organic polymers (POPs), especially conjugated microporous or porous polymers (CMPs or CPPs), have received increasing attention due to their remarkable and designable properties, such as chemical and thermal stability, tunable band gap, large surface area and adjustable porosity due to their porous nature. All these features make them highly attractive systems for applications in different fields, including gas storage and separation, energy storage, water purification, photoelectricity, chemosensors, organic electronics and heterogenous catalysis (e.g., photocatalysis) [235]. A wide range of π-conjugated organic molecules have been used to build CMPs and CCPs, including nitrogen-, oxygen- and sulfur-containing heteroaromatic moieties such as tetrathienoanthracenes (TTA), naphthodithiophenes and benzodithiophenes. In 2021, a 3D π-conjugated microporous polymer **243** (Figure 25), containing the **BDT-1**-based tetrathienoanthracene (TTA), was synthesized through a bottom-up approach, and used as a heterogeneous organocatalyst for visible light-promoted organic reactions [236]. The great photoredox features along with its excellent stability and mesoporous structure made the polymer **243** a highly efficient (80–98% yield for 29 examples) and reusable (10–12 times) heterogenous photocatalytic system for model organic transformations, including the oxidative C–C bond formation between *N*-aryltetrahydroisoquinolines and nitroalkanes or ketones, and the coupling of phenylenediamines with aldehydes towards the synthesis of benzimidazoles.

In 2023, the TTA framework was also employed as a sulfur-rich and π-conjugated skeleton to design and synthesize star-type 3D CPPs that represented promising candidates for photocatalytic CO_2_ conversion [237].

The polymer arising from the Sonogashira–Hagihara coupling (SHC) between **Br-TTA** (Figure 25) with the more flexible ethynylphenyl methane structure (**EtPhM**, Figure 25) exhibited a larger specific surface area, a better CO_2_ adsorption capacity, and a lower energy barrier for the rate-determining step of CO_2_ photoconversion than those observed for the more rigid pyrene-based polymer formed by SHC between **Br-TTA** and **EtPy** (Figure 25). Indeed, the **EtPhM**-based polymer provided a higher CO evolution rate and selectivity (up to 322.05 μmol g^−1^ h^−1^ and 99.26%, respectively).

#### 4.8.2. BDT-Based Multifunctional Materials

**BDT-1** derivatives **244** (Figure 25), differing in the position of the nitrogen atom in the pyridine rings, were identified as multifunctional materials with peculiar self-assembly behavior [238]. In particular, compound **244a**, with nitrogen in the *para* position, self-assembled into a columnar liquid-crystalline phase and exhibited significant, red-shifted absorption and emission peaks due to enhanced conjugation. Further, **244a** also formed gels in different organic solvents, and when doped with L-tartaric acid, gave a gel with intensified yellow emission, suitable for white light-emitting diodes (WLEDs) production. Moreover, the protonation of pyridine in **244** enabled acid–base vapor detection, and both compounds exhibited pronounced acidochromic behavior, making them promising indicators for acid–base sensing in the film states.

## 5. Conclusions and Perspectives

This review underscores, for the first time, the significance of isomeric benzodithiophene (**BDT**), benzodifuran (**BDF**) and benzodiselenophene (**BDS**) analogs of phenantrene as key members of chalcogen-containing tricyclic β-fused systems. Their distinctive angular architecture imparts properties markedly different from those of their anthracene-like counterparts, while the spatial arrangement of heteroatoms within these frameworks emerges as a critical determinant of structure–property relationships. These features influence not only the behavior of individual molecules but also their performance within polymeric architectures, where the nature of the heteroatom plays a pivotal role in modulating electronic characteristics.

**BDT** derivatives emerge as the most prominent class discussed in this review, owing to the breadth and versatility of synthetic methodologies developed for their construction. Robust and modular strategies for assembling **BDT** frameworks have enabled extensive structural diversification, facilitating downstream functionalization and integration into advanced material architectures. Moreover, **BDT** scaffolds are typically obtained as stable frameworks amenable to post-synthetic modification, underscoring their higher stability and synthetic flexibility than BDF derivatives. Indeed, most reported protocols for synthesizing **BDF** derivatives typically afford a single, well-defined compound and frequently yield pre-functionalized cores, thereby limiting opportunities for structural diversification. On the other hand, furan has recently emerged as a privileged building block among group 16 heterocycles, driven by its biodegradability and renewable sourcing [239]. Thus, the development of innovative synthetic strategies for furan-fused π-conjugated systems represents a promising approach toward sustainable materials design. The development of synthetic strategies for **BDS** derivatives is still at an early stage, with only a limited number of reported procedures that typically afford low yields. Nevertheless, efficient post-synthetic functionalization of the **BDS** core—such as functionalization of α-positions of selenium atoms and subsequent Pd-catalyzed coupling reactions—has been successfully demonstrated, highlighting significant opportunities for further development.

Among the applications reported for **BDT**, **BDF**, and **BDS** derivatives, optoelectronics stands out as the most extensively explored field, with numerous examples of significant relevance. The synthetic flexibility and stability of **BDT** frameworks have enabled a wide range of advanced device architectures, including OLEDs, OFETs, and photovoltaic systems. This versatility is further enhanced by the ease of oligomerization and polymerization of the **BDT** scaffold, which affords high-performance **BDT**-based polymers as semiconductors in bulk heterojunction organic solar cells. Controlled functionalization strategies allow for fine-tuning of electronic properties, reinforcing the pivotal role of **BDT** scaffolds in bridging molecular design with practical implementation. Looking ahead, the implementation of more sustainable synthetic procedures—aligned with the principles of green chemistry—will be essential to ensure that **BDT** derivatives become truly competitive and viable for large-scale applications.

Although less developed, **BDT** and **BDF** derivatives exhibit promising biological potential, functioning as cytotoxic agents and fluorophores for NIR-II bioimaging when appropriately engineered. This demonstrates that compounds traditionally confined to optoelectronic applications can gain attention for their potential roles in biological systems. Such emerging trends exemplify the growing interplay between materials science and life sciences, opening avenues for multifunctional platforms that integrate electronic performance with biomedical relevance.

On the other hand, **BDS** frameworks—owing to their high polarizability and tunable electronic characteristics—represent attractive candidates for future optoelectronic applications. Post-synthetic functionalization offers significant opportunities for tailoring electronic structures, paving the way for advanced materials in organic semiconductors, photovoltaic devices, and nonlinear optical systems. Expanding the synthetic toolbox to improve yields and structural diversity will be essential to fully exploit the potential of **BDS** derivatives in next-generation technologies.

## Figures and Tables

**Table 1:** Mechanistic Stages of **BDT**/**BDS** electropolymerization.

Stage	Electrochemical/Chemical Process	Structural Evolution	Kinetic Consequence
1. Activation	$M\to M^{+\cdot }$ + e^−^	Generation of paramagnetic radical cation	Current response proportional to monomer concentration
2. Aggregation	$2M^{+\cdot }⇌[M_{2}{]}_{\left(\pi \right)}^{2+}$	Formation of face-to-face π-dimer stack	Stabilization of charge; pre-alignment for coupling
3. Coupling	$[M_{2}{]}_{\left(\pi \right)}^{2+}\to (M-M{)}_{\left(\sigma \right)}^{2+}$	Formation of C-C σ-bond; loss of aromaticity	Rate-determining step; sensitive to steric hindrance
4. Elimination	$(M-M{)}_{\left(\sigma \right)}^{2+}\to M_{2}+2H^{+}$	Re-aromatization; proton release	Irreversible step; potential for acid-catalysed degradation
5. Growth	$M_{2}\to M_{2}^{+\cdot }\ldots \to P_{solid}$	Extension of chain; precipitation	Nucleation and growth of solid film on electrode

**Table 2:** Electrochemical parameters of **BDT** and **BDS**.

Compound	Substituent	$E_{ox}^{onset}$ (V vs. Fc|Fc^+^)	HOMO (eV)	LUMO (eV)	Band Gap (*E*_g_)
**BDT**	H	+0.95 (*E*_pa_)	−5.60	−2.00	3.60
**BDS**	H	+0.89 (*E*_pa_)	−5.50	−2.00	3.50
**BDTe**	H	+0.48 (*E*_pa_)	−5.10	−2.00	3.10
**BDT**-Alkoxy	OR (e.g., ethylhexyloxy)	~+0.50	~−5.16	~−3.47	~1.69
**BDT**-Thienyl	Thiophene R	~+0.80	~−5.45	~−3.55	~1.90
**BDS**-Thienyl	Thiophene R	~+0.70	~−5.35	~−3.60	~1.75

## Data Availability

No new data were created or analyzed in this work. Data sharing is not applicable to this article.
